# Supplementary material for: Comprehensive analysis of genes associated with necroptosis and pyroptosis in intestinal ischemia-reperfusion injury
Source: PLoS One. 2025 Dec 16;20(12):e0338420. doi: 10.1371/journal.pone.0338420 (PMC12707633; doi:10.1371/journal.pone.0338420)
Supplement: S1 Table — (DOCX) [file pone.0338420.s001.docx]

################ Step 1: Data Acquisition and Preprocessing ###########

# The following six lines are code for installing the corresponding packages

if(!requireNamespace("BiocManager",quietly = T))

install.packages("BiocManager")

if(!requireNamespace("GEOquery",quietly = T))

BiocManager::install("GEOquery")

if(!requireNamespace("limma",quietly = T))

BiocManager::install("limma",force = TRUE)

if(!requireNamespace("tidyverse",quietly = T))

install.packages("tidyverse",force = TRUE)

library(tidyverse)

library(limma)

library(GEOquery)

# Data acquisition and preprocessing

if (!file.exists("GSE96733_eSet.Rdata")) {

GEO_file <- getGEO('GSE96733',# Series to download

# destdir = 'E:/GEOdoc/GSE64634',# Set file save path

getGPL = T # Download platform file

)

save(GEO_file, file = "GSE96733_eSet.Rdata")# Save downloaded file in processable format

}

load("GSE96733_eSet.Rdata")# Load the formatted and saved data

# Extract the required parts from the downloaded data

GEO_file[[1]]# Extract the first data from GEO_file

exp <- exprs(GEO_file[[1]])# Extract sample gene expression matrix from data

plate <- fData(GEO_file[[1]])# Extract platform information from data

clinical <- pData(GEO_file[[1]])# Extract sample clinical information from data (e.g., age, gender, survival status, etc.)

write.csv(clinical, "GSE96733_clinical.csv")

# Download matrix file and platform file for GSE96733 from GEO database, replace gene probes with gene names to get file GSE96733.csv

GSE96733 <- read.csv("GSE96733.csv", row.names = 1)

# Extract 4 sham operation samples and 4 II/R 3-hour samples

GSE_1 <- GSE96733[,c(1,2,3,4,9,10,11,12)]

write.csv(GSE_1, file = "GSE_1.CSV")

# Extract 4 sham operation samples and 4 II/R 6-hour samples as validation set

GSE_2 <- GSE96733[,c(1,2,3,4,17,18,19,20)]

write.csv(GSE_2, file = "GSE_2.CSV")

# Obtain mouse necroptosis, pyroptosis, and ferroptosis genes from NCBI database

## Obtain validation set expression matrix

library(tidyverse)

library(limma)

library(GEOquery)

# Step 1: Data acquisition and preprocessing

if (!file.exists("GSE37013_eSet.Rdata")) {

GEO_file <- getGEO('GSE37013',# Series to download

# destdir = 'E:/GEOdoc/GSE64634',# Set file save path

getGPL = T # Download platform file

)

save(GEO_file, file = "GSE37013_eSet.Rdata")# Save downloaded file in processable format

}

load("GSE37013_eSet.Rdata")# Load the formatted and saved data

# Extract the required parts from the downloaded data

GEO_file[[1]]# Extract the first data from GEO_file

exp <- exprs(GEO_file[[1]])# Extract sample gene expression matrix from data

plate <- fData(GEO_file[[1]])# Extract platform information from data

clinical <- pData(GEO_file[[1]])# Extract sample clinical information from data (e.g., age, gender, survival status, etc.)

write.csv(clinical, "GSE37013_clinical.csv")

# Check data distribution

exp <- as.matrix(exp)

boxplot(exp)

### Data integration using downloaded platform data

ID <- data.frame(ID_REF = plate$ID, Gene_Symbol = plate$`Symbol`)# Extract ID column and SYMBOL column from platform file

ID$Gene_Symbol <-data.frame(sapply(ID$Gene_Symbol,function(x)unlist(strsplit(x,"///"))[1]),stringsAsFactors=F)[,1]

exp <- as.data.frame(exp)

exp$ID_REF <- rownames(exp)

exp <- merge(exp, ID, by='ID_REF')# Use merge() function to match probe ids in dat1 with chip platform probe ids, merge into dat1

exp[, grep("Gene_Symbol", colnames(exp))] <- trimws(exp[, grep("Gene_Symbol", colnames(exp))])# Remove leading/trailing spaces from data

exp[exp==""] <- NA# Assign NA to blank values

exp <- na.omit(exp)# Delete data with missing GENE_SYMBOL

exp <- as.data.frame(exp)

write.csv(exp, "GSE37013_exp.csv")

exp <- read.csv("GSE37013_exp.csv", row.names = 1)

exp[exp < 0] <- 0

table(duplicated(exp$Gene_Symbol))# Check number of duplicates

# Take average of duplicate genes

exp_1 <- avereps(exp, ID = exp$Gene_Symbol)

exp_1 <- as.data.frame(exp_1)

rownames(exp_1) <- exp_1$Gene_Symbol# Add row names

exp_1 <- exp_1[,-c(1,ncol(exp_1))]# Remove redundant column data

write.csv(exp_1, "GSE37013_exp_average.csv")# Save as csv format

## Extract as validation set

GSE_4 <- read.csv("GSE37013_exp_average.csv", row.names = 1)

GSE_5 <- GSE_4[,c(1:7,15:21)]

write.csv(GSE_5, "GSE_5.csv")

####### GSE232246 Download and Processing

setwd("./GSE232246")

# Load necessary packages

library(biomaRt)

library(dplyr)

library(tidyr)

# Read data

data <- read.csv("GSE232246_FPKM.csv", header = TRUE, stringsAsFactors = FALSE)

# Check data structure

head(data)

dim(data)

# Use biomaRt for ID conversion

# Connect to mouse Ensembl database

ensembl <- useMart("ensembl", dataset = "mmusculus_gene_ensembl")

# Extract Track_id (remove version number if present)

track_ids <- gsub("\\..*", "", data$Track_id)

# Get gene symbols

gene_info <- getBM(

attributes = c("ensembl_transcript_id", "external_gene_name"),

filters = "ensembl_transcript_id",

values = track_ids,

mart = ensembl

)

# Merge gene names to original data

colnames(gene_info) <- c("Track_id", "Gene_Symbol")

data_with_gene <- merge(data, gene_info, by = "Track_id", all.x = TRUE)

# Check matching status

unmatched_na <- sum(is.na(data_with_gene$Gene_Symbol))

unmatched_blank <- sum(data_with_gene$Gene_Symbol == "", na.rm = TRUE)

cat("Number of transcripts without gene name (NA):", unmatched_na, "\n")

cat("Number of transcripts with blank gene name:", unmatched_blank, "\n")

cat("Total number of transcripts:", nrow(data_with_gene), "\n")

# Remove rows without gene name and blank gene names

data_clean <- data_with_gene %>%

filter(!is.na(Gene_Symbol) & Gene_Symbol != "")

cat("Number of genes retained after cleaning:", nrow(data_clean), "\n")

# Take average of duplicate genes

# Extract numeric columns (sample columns)

sample_cols <- colnames(data_clean)[2:7] # GSM7324489 to GSM7324491, GSM7324486 to GSM7324488

# Group by gene name and calculate mean

data_avg <- data_clean %>%

group_by(Gene_Symbol) %>%

summarise(across(all_of(sample_cols), \(x) mean(x, na.rm = TRUE))) %>%

ungroup()

# Check processed data

cat("Final number of genes:", nrow(data_avg), "\n")

head(data_avg)

# Optional: Save processed data

write.csv(data_avg, "GSE232246_FPKM_gene_avg.csv", row.names = FALSE)

# Check duplicate gene processing

duplicate_genes <- data_clean %>%

count(Gene_Symbol) %>%

filter(n > 1) %>%

arrange(desc(n))

cat("Number of genes with duplicate transcripts:", nrow(duplicate_genes), "\n")

if(nrow(duplicate_genes) > 0) {

cat("Top 10 genes with most duplicates:\n")

print(head(duplicate_genes, 10))

}

# Data quality check

cat("\nData summary:\n")

summary(data_avg[, sample_cols])

# Check expression distribution

cat("\nExpression statistics for each sample:\n")

apply(data_avg[, sample_cols], 2, function(x) {

c(Mean = mean(x), Median = median(x), SD = sd(x), Min = min(x), Max = max(x))

})

############### Step 2: Differential Expression ############

# Load necessary packages

library(tidyverse)

library(limma) # For differential expression analysis

library(dplyr) # For data processing

library(ggplot2)

library(pheatmap)

library(ggrepel)

# Step 1: Read data

GSE_1 <- read.csv("GSE_1.csv", row.names = 1, check.names = FALSE)

# row.names=1 sets first column as row names (gene names)

# check.names=FALSE preserves numbers at beginning of column names (like GSM numbers)

# Step 2: Create grouping information

# First 4 columns are control group (Control), last 4 columns are intestinal ischemia-reperfusion injury group (IRI)

group <- factor(c(rep("Control", 4), rep("IRI", 4)),

levels = c("Control", "IRI")) # Set control group as baseline

group

# Step 3: Construct design matrix

design <- model.matrix(~0 + group) # Create design matrix without intercept

colnames(design) <- c("Control", "IRI") # Rename columns to "Control" and "IRI" for subsequent comparisons

# Step 4: Linear model fitting

fit <- lmFit(GSE_1, design)# Fit each gene's expression value to linear model

# Step 5: Set contrast matrix (compare IRI group vs Control group)

contrast_matrix <- makeContrasts(IRI_vs_Control = IRI - Control, levels = design)

fit2 <- contrasts.fit(fit, contrast_matrix)# Set specific comparison group (IRI vs Control)

# Step 6: Bayesian test

fit2 <- eBayes(fit2)# Apply empirical Bayes smoothing, improve variance estimation, enhance reliability of small sample data analysis

# Step 7: Extract differential analysis results

DEGs_results <- topTable(fit2, coef = "IRI_vs_Control",

number = Inf, adjust.method = "BH")

# coef: Specify comparison group

# number: Output all genes (Inf means all)

# adjust.method: Use BH method to adjust p-values (FDR)

# Step 8: Add gene column

DEGs_results$Gene <- rownames(DEGs_results)

# Step 9: Filter differentially expressed genes (DEGs)

DEGs <- DEGs_results %>%

filter(abs(logFC) >= 1 & adj.P.Val < 0.05) %>%

arrange(desc(abs(logFC))) # Sort by |logFC| descending

# Step 10: Output results

# Output complete results for all genes

write.csv(DEGs_results, "all_gene_results.csv", row.names = FALSE)# Contains complete analysis results for all genes, logFC: log2 fold change of IRI group vs Control group, adj.P.Val: BH-adjusted p-value (FDR), AveExpr: average expression of all samples, t: t statistic, P.Value: raw p-value, B: B statistic (empirical Bayes statistic)

# Output differentially expressed genes

write.csv(DEGs, "DEGs_results.csv", row.names = FALSE)# Contains only differentially expressed genes meeting threshold, sorted by |logFC| descending, facilitating viewing of most significant differential genes

DEGs <- read.csv("DEGs_results.csv")

# Step 11: Results overview

cat("Number of differentially expressed genes:", nrow(DEGs), "\n")

cat("Number of upregulated genes:", sum(DEGs$logFC > 0), "\n")

cat("Number of downregulated genes:", sum(DEGs$logFC < 0), "\n")

############ Step 3: Volcano Plot and Heatmap Drawing ########

######## Volcano Plot Drawing ##########

# Load necessary packages

library(ggplot2) # For plotting

library(ggrepel) # For intelligent label avoidance

library(pheatmap) # For drawing heatmaps

library(dplyr) # For data processing

library(RColorBrewer) # Provides color schemes

packageVersion("ggplot2")

# 1. Read differentially expressed gene data

all_degs <- read.csv("all_gene_results.csv", header = TRUE)

# 2. Data preprocessing

# Add significance marker column: according to common criteria (|logFC|>1 & adj.P.Val<0.05)

all_degs <- all_degs %>%

mutate(

Significance = case_when(

logFC > 1 & adj.P.Val < 0.05 ~ "Up-regulated",

logFC < -1 & adj.P.Val < 0.05 ~ "Down-regulated",

TRUE ~ "Not significant"

),

# Create column for label display: only mark significant genes

Label = ifelse(abs(logFC) > 1 & adj.P.Val < 0.05, Gene, "")

)

# 3. Create basic volcano plot

volcano_plot <- ggplot(all_degs, aes(x = logFC, y = -log10(adj.P.Val),

color = Significance, label = Label)) +

geom_point(alpha = 0.6, size = 1.5) + # Draw point plot, set transparency 0.6 and size 1.5

scale_color_manual(values = c("Down-regulated" = "blue",

"Not significant" = "gray",

"Up-regulated" = "red")) + # Set colors

geom_hline(yintercept = -log10(0.05), linetype = "dashed", color = "black") + # P-value threshold line

geom_vline(xintercept = c(-1, 1), linetype = "dashed", color = "black") + # logFC threshold lines

labs(x = "log2 Fold Change", y = "-log10(Adjusted P-value)", # Axis labels

title = "Volcano Plot of DEGs (Control vs IRI)",

subtitle = "Significant genes: |logFC| > 1 & adj.P.Val < 0.05") +

theme_minimal(base_size = 12) + # Use minimal theme, set base font size

theme(

legend.position = "top", # Legend position at top

plot.title = element_text(hjust = 0.5, face = "bold"), # Center title and bold

plot.subtitle = element_text(hjust = 0.5), # Center subtitle

# Use linewidth instead of deprecated size parameter

panel.grid.major = element_line(color = "grey90", linewidth = 0.2), # Set grid lines

panel.grid.minor = element_blank() # Remove minor grid lines

)

# 4. Add labels - only mark top 10 most significant genes

# Selection criteria: sort by adjusted p-value, then take top 10 by absolute logFC

top_genes <- all_degs %>%

filter(Significance != "Not significant") %>% # Only select significant genes

arrange(adj.P.Val, desc(abs(logFC))) %>% # First sort by p-value, then by absolute logFC

head(10) # Take top 10

# 5. Add labels to volcano plot

volcano_plot_labeled <- volcano_plot +

geom_text_repel(

data = top_genes, # Only use top genes data

aes(label = Gene), # Display gene names

size = 3.5, # Label font size

box.padding = 0.5, # Padding around labels

max.overlaps = 20, # Maximum allowed overlap

segment.color = "grey50", # Connection line color

segment.size = 0.3, # Connection line thickness

min.segment.length = 0.1 # Minimum connection line length

) +

scale_y_continuous(expand = expansion(mult = c(0.05, 0.15))) # Expand y-axis range to make space for labels

# 6. Save volcano plot

ggsave("volcano_plot.pdf", plot = volcano_plot_labeled,

width = 10, height = 8, dpi = 300, bg = "white")

####### Heatmap Drawing ########

# Load necessary packages

library(pheatmap)

library(dplyr)

library(RColorBrewer)

# Step 1: Read differentially expressed gene results

degs <- read.csv("DEGs_results.csv", header = TRUE)

# Filter significant differentially expressed genes (|logFC| > 1 & adj.P.Val < 0.05)

significant_genes <- degs %>%

filter(abs(logFC) > 1 & adj.P.Val < 0.05) %>%

arrange(desc(abs(logFC))) # Sort by absolute logFC descending

# Selected key genes

top_genes <-significant_genes

sig_gene_names <- top_genes$Gene

# Step 2: Read original expression matrix

expression_matrix <- read.csv("GSE_1.CSV", header = TRUE, row.names = 1)

# Step 3: Extract expression data for key genes

heatmap_data <- expression_matrix[rownames(expression_matrix) %in% sig_gene_names, ]

# Check and handle missing genes

missing_genes <- setdiff(sig_gene_names, rownames(heatmap_data))

if (length(missing_genes) > 0) {

message("The following genes are missing from expression matrix: ", paste(missing_genes, collapse = ", "))

# Update gene list, only keep existing genes

sig_gene_names <- sig_gene_names[!sig_gene_names %in% missing_genes]

}

# Sort genes by logFC (upregulated first, then downregulated)

gene_order <- top_genes %>%

filter(Gene %in% sig_gene_names) %>%

arrange(desc(logFC)) # Descending order: upregulated genes first, downregulated last

heatmap_data <- heatmap_data[gene_order$Gene, ]

# Step 4: Data standardization (Z-score)

scaled_data <- t(scale(t(heatmap_data)))

# Step 5: Create sample grouping annotation

sample_groups <- data.frame(

Group = rep(c("Control", "IRI"), each = 4),

row.names = colnames(scaled_data)

)

# Step 6: Set color scheme

group_colors <- list(Group = c(Control = "#1F77B4", IRI = "#FF7F0E")) # Blue and orange

color_palette <- colorRampPalette(rev(brewer.pal(n = 11, name = "RdBu")))(100)

# Step 7: Draw heatmap

heatmap_plot <- pheatmap(

scaled_data,

scale = "none",

color = color_palette,

border_color = "gray60", # Add light gray border to improve readability

#cellwidth = 15, # Fixed cell width

#cellheight = 15, # Fixed cell height

# Row settings

show_rownames = F,

cluster_rows = FALSE, # Do not cluster rows

fontsize_row = 10, # Increase row name font size

# Column settings

show_colnames = T,

cluster_cols = FALSE, # Do not cluster columns

fontsize_col = 11, # Column name font size

angle_col = 90, # Column name tilt 45 degrees

# Grouping annotation

annotation_col = sample_groups,

annotation_colors = group_colors,

# Graphic settings

main = "Expression Heatmap of DEGs\n(Control vs IRI)",

treeheight_row = 0, # Do not show row clustering tree

treeheight_col = 0, # Do not show column clustering tree

# Legend settings

legend = TRUE,

annotation_legend = TRUE

)

# Step 8: Save heatmap

# Save as PDF

pdf("Top_DEGs_heatmap.pdf", width = 8, height = 6)

grid::grid.newpage()

grid::grid.draw(heatmap_plot$gtable)

dev.off()

# Save as PNG

png("Top_DEGs_heatmap.png", width = 8, height = 6, units = "in", res = 300)

grid::grid.newpage()

grid::grid.draw(heatmap_plot$gtable)

dev.off()

message("Heatmap saved as Top_DEGs_heatmap.pdf and Top_DEGs_heatmap.png")

# Combine volcano plot and heatmap #

########## Combine Volcano Plot and Heatmap (side by side layout) ##########

# Load necessary packages

library(cowplot)

library(ggplotify) # For converting pheatmap objects to ggplot objects

# Step 1: Convert heatmap to ggplot object

heatmap_ggplot <- as.ggplot(heatmap_plot)

# Step 2: Create main title for combined plot

main_title <- ggdraw() +

draw_label(

"",

fontface = "bold",

size = 16,

x = 0.5,

hjust = 0.5

)

# Step 3: Add panel labels for each plot

# Volcano plot label (top left)

volcano_label <- ggdraw() +

draw_label("A",

x = 0.02, hjust = 0,

size = 24, fontface = "bold")

# Heatmap label (top left)

heatmap_label <- ggdraw() +

draw_label("B",

x = 0.02, hjust = 0,

size = 24, fontface = "bold")

# Step 4: Combine volcano plot and its label

volcano_with_label <- plot_grid(

volcano_label,

volcano_plot_labeled,

ncol = 1,

rel_heights = c(0.08, 1) # Label occupies 8%, plot occupies 92%

)

# Step 5: Combine heatmap and its label

heatmap_with_label <- plot_grid(

heatmap_label,

heatmap_ggplot,

ncol = 1,

rel_heights = c(0.08, 1) # Label occupies 8%, plot occupies 92%

)

# Step 6: Combine two graphs side by side

combined_plot <- plot_grid(

main_title, # Main title

# Side by side arranged graphs

plot_grid(

volcano_with_label,

heatmap_with_label,

nrow = 1, # Single row layout

rel_widths = c(1, 1.2) # Volcano plot width 1, heatmap width 1.2 (heatmap usually needs more space)

),

# Overall layout

nrow = 2,

rel_heights = c(0.07, 1), # Title height 7%, graph height 93%

align = "h", # Horizontal alignment

axis = "tb" # Top and bottom alignment

)

# Step 7: Save combined plot (side by side layout)

# PDF format

ggsave("combined_plot_side_by_side.pdf",

plot = combined_plot,

width = 16, # Wider dimensions for side by side layout

height = 8, # Height can be less than vertical layout

dpi = 300,

bg = "white")

# PNG format

ggsave("combined_plot_side_by_side.png",

plot = combined_plot,

width = 16,

height = 8,

dpi = 300,

bg = "white")

# TIFF format

ggsave("combined_plot_side_by_side.tiff", plot = combined_plot,

dpi = 300, # Resolution (DPI, journals usually require ≥300)

width = 16, height = 8,

units = "in", # Unit (pixels)

compression = "lzw")# Compression algorithm (reduce file size)

message("Side by side combined plot saved as combined_plot_side_by_side.pdf and combined_plot_side_by_side.png")

######## Step 4: Obtain Death-Related Differential Genes and Draw Venn Diagram ######

install.packages("VennDiagram")

install.packages("gridExtra")

# Load necessary packages

library(VennDiagram) # For drawing Venn diagrams

library(dplyr) # For data processing

library(grid) # For graphic layout

library(gridExtra) # For combining graphics

# Read differential gene data

degs <- read.csv("DEGs_results.csv", stringsAsFactors = FALSE)

degs_genes <- degs$Gene

head(degs_genes)

# Read necroptosis gene set (168 genes)

necroptosis <- read.csv("gene_necroptosis.csv", stringsAsFactors = FALSE)

necroptosis_genes <- necroptosis$Gene

head(necroptosis_genes)

# Read pyroptosis gene set (299 genes)

pyroptosis <- read.csv("gene_pyroptosis.csv", stringsAsFactors = FALSE)

pyroptosis_genes <- pyroptosis$Gene

head(pyroptosis_genes)

# Calculate intersections

necroptosis_intersect <- intersect(degs_genes, necroptosis_genes)

pyroptosis_intersect <- intersect(degs_genes, pyroptosis_genes)

# Create output directory

dir.create("results", showWarnings = FALSE)

# Save intersection gene results

write.csv(data.frame(Gene = necroptosis_intersect),

"results/necroptosis_intersection.csv", row.names = FALSE)

write.csv(data.frame(Gene = pyroptosis_intersect),

"results/pyroptosis_intersection.csv", row.names = FALSE)

# Draw necroptosis Venn diagram

venn_necro <- draw.pairwise.venn(

area1=length(degs_genes),

area2 = length(necroptosis_genes),

cross.area = length(necroptosis_intersect),

category = c("DEGs", "NRGs"),

fill = c("#1f78b4", "#33a02c"),

alpha = 0.5,

cat.pos = c(0, 0),

cat.dist = c(0.05, 0.05),

cat.cex = 1.5,

cex = 1.8,

ext.text = FALSE,

ind = TRUE

)

# Draw pyroptosis Venn diagram

venn_pyro <- draw.pairwise.venn(

area1 = length(degs_genes),

area2 = length(pyroptosis_genes),

cross.area = length(pyroptosis_intersect),

category = c("DEGs", "PRGs"),

fill = c("#1f78b4", "orange"),

alpha = 0.5,

cat.pos = c(0, 0),

cat.dist = c(0.05, 0.05),

cat.cex = 1.5,

cex = 1.8,

ext.text = FALSE,

ind = TRUE

)

# Save as PDF

pdf("results/cell_death_venn.pdf", width = 12, height = 6)

grid.arrange(gTree(children = venn_necro),

gTree(children = venn_pyro),

ncol = 2)

dev.off()

# Save as PNG

png("results/cell_death_venn.png", width = 1200, height = 600, res = 150)

grid.arrange(gTree(children = venn_necro),

gTree(children = venn_pyro),

ncol = 2)

dev.off()

# Print result summary

cat("Necroptosis intersection results:\n")

cat("Number of DEGs:", length(degs_genes), "\n")

cat("Number of necroptosis genes:", length(necroptosis_genes), "\n")

cat("Number of intersection genes:", length(necroptosis_intersect), "\n")

cat("Intersection genes:", paste(necroptosis_intersect, collapse = ", "), "\n\n")

cat("Pyroptosis intersection results:\n")

cat("Number of DEGs:", length(degs_genes), "\n")

cat("Number of pyroptosis genes:", length(pyroptosis_genes), "\n")

cat("Number of intersection genes:", length(pyroptosis_intersect), "\n")

cat("Intersection genes:", paste(pyroptosis_intersect, collapse = ", "))

######## Obtain Death-Related Differential Genes ########

P_gene <- read.csv("./results/pyroptosis_intersection.csv")

N_gene <- read.csv("./results/necroptosis_intersection.csv")

P_N_gene <- bind_rows(N_gene,P_gene)

duplicated(P_N_gene)# Check duplicates

P_N_gene <- P_N_gene[duplicated(P_N_gene),]

write.csv(P_N_gene, "./results/P_N_gene.csv", row.names = FALSE)

# Draw hub gene Venn diagram

N_gene <- N_gene$Gene

P_gene <- P_gene$Gene

venn_hub <- draw.pairwise.venn(

area1 = length(N_gene),

area2 = length(P_gene),

cross.area = length(P_N_gene),

category = c("DENRGs", "DEPRGs"),

fill = c("#E59CC4", "yellow"),

alpha = 0.5,

cat.pos = c(0, 0),

cat.dist = c(0.05, 0.05),

cat.cex = 1.5,

cex = 1.8,

ext.text = FALSE,

ind = TRUE

)

# Save as PDF

pdf("results/Hub_venn.pdf", width = 12, height = 6)

grid.arrange(gTree(children = venn_hub),

ncol = 1)

dev.off()

# Save as PNG

png("results/Hub_venn.png", width = 1200, height = 600, res = 150)

grid.arrange(gTree(children = venn_hub),

ncol = 1)

dev.off()

###### Obtain Death-Related Differential Gene Expression Matrix ######

# Load necessary packages

library(dplyr) # For data processing

library(tibble) # For handling row name conversion

# Step 1: Read DCDEG gene list

# Read CSV file containing target gene names

DCDEG_genes <- read.csv("P_N_gene.csv", stringsAsFactors = FALSE)

# Extract gene name vector

target_genes <- DCDEG_genes$Gene

cat("Found", length(target_genes), "target genes\n")

# Step 2: Read expression profile matrix

# Read expression profile CSV file (first column is gene names, first row is sample names)

# row.names = 1 sets first column as row names (gene names)

exp_matrix <- read.csv("GSE_1.csv", row.names = 1, check.names = FALSE)

# Check expression profile structure

cat("\nExpression matrix dimensions:", dim(exp_matrix), "\n")

cat("First 5 genes:", head(rownames(exp_matrix), 5), "\n")

# Step 3: Gene name matching

# Create matching gene names (expression profile gene names)

exp_genes <- rownames(exp_matrix)

# Find positions of target genes in expression profile

matched_indices <- match(target_genes, exp_genes)

# Count matching results

matched_genes <- rownames(exp_matrix)[matched_indices[!is.na(matched_indices)]]

unmatched <- target_genes[is.na(matched_indices)]

cat("\nSuccessfully matched genes:", length(matched_genes), "/", length(target_genes))

cat("\nUnmatched genes:", ifelse(length(unmatched) > 0, paste(unmatched, collapse = ", "), "None"))

# Step 4: Extract target gene expression matrix

# Extract expression data for matched genes

deg_expression <- exp_matrix[matched_indices[!is.na(matched_indices)], ]

# Add original gene names (from DCDEG) as new column

deg_expression <- deg_expression %>%

rownames_to_column(var = "ExpMatrix_GeneName") %>% # Keep original gene names from expression profile

mutate(DCDEG_Gene = DCDEG_genes$Gene[!is.na(matched_indices)]) %>% # Add gene names from DCDEG

relocate(DCDEG_Gene, .before = ExpMatrix_GeneName) # Move DCDEG gene name column to front

deg_expression <- deg_expression[,-2]

# Step 5: Save results

# Save extracted expression matrix

write.csv(deg_expression, "N_P_expression_Matrix.csv", row.names = FALSE)

# Step 6: Output final results

cat("\n\nResults saved as DCDEG_Expression_Matrix.csv")

cat("\nFile contains", nrow(deg_expression), "genes in", ncol(deg_expression)-2, "sample expression data\n")

# Display expression situation of first 5 genes

cat("\nExpression summary of first 5 genes:\n")

print(head(deg_expression, 5))

# Draw violin plot of death-related differential gene expression levels

# Load necessary packages

library(ggplot2) # Core plotting package

library(tidyr) # Data tidying

library(dplyr) # Data processing

library(RColorBrewer) # Color configuration

# 1. Read expression matrix data -----------------------------------------------------

expr_data <- read.csv("N_P_expression_Matrix.csv", check.names = FALSE)

# 2. Data preprocessing ----------------------------------------------------------

# Convert to long format (gene-sample-expression value)

long_data <- expr_data %>%

pivot_longer(

cols = -DCDEG_Gene,

names_to = "Sample",

values_to = "Expression"

)

# Add grouping information (first 4 samples are Control, last 4 are IRI)

long_data <- long_data %>%

mutate(

Group = ifelse(Sample %in% names(expr_data)[2:5], "Control", "IRI"),

Group = factor(Group, levels = c("Control", "IRI")) # Set factor order

)

# 3. Create violin plot -------------------------------------------------------

violin_plot <- ggplot(long_data, aes(x = DCDEG_Gene, y = Expression, fill = Group)) +

# Violin layer

geom_violin(

scale = "width", # Uniform violin width

trim = TRUE, # Trim tails

position = position_dodge(0.8),

alpha = 0.7, # Transparency

width = 0.75 # Width adjustment

) +

# Boxplot layer

geom_boxplot(

width = 0.15, # Boxplot width

position = position_dodge(0.8),

outlier.size = 0.5, # Outlier point size

show.legend = FALSE

) +

# Scatter layer

geom_jitter(

size = 1.0, # Point size

alpha = 0.6, # Transparency

position = position_jitterdodge(jitter.width = 0.15, dodge.width = 0.8),

show.legend = FALSE

)

# 4. Optimize graphic appearance --------------------------------------------------------

optimized_plot <- violin_plot +

# Color scheme (Control: blue, IRI: red)

scale_fill_manual(values = c("#3498DB", "#E74C3C")) +

# English labels and titles

labs(

title = "DCDEGs Expression in Mouse Intestinal IRI Model",

#subtitle = "",

x = "DCDEGs",

y = "Normalized Expression (log2)",

fill = "Group"

) +

# Theme customization

theme_classic(base_size = 14) +

theme(

plot.title = element_text(hjust = 0.5, face = "bold", size = 16), # Center title and bold

plot.subtitle = element_text(hjust = 0.5, size = 14), # Center subtitle

axis.text.x = element_text(angle = 90, hjust = 1, face = "italic"), # Italic gene names

axis.title = element_text(face = "bold", size = 14), # Bold axis titles

axis.text = element_text(size = 12), # Axis text size

legend.position = "top", # Legend at top

legend.title = element_text(face = "bold"), # Bold legend title

legend.text = element_text(size = 12), # Legend text size

panel.grid.major.y = element_line(color = "grey90") # Horizontal grid lines

) +

scale_y_continuous(expand = c(0, 0)) # Y-axis starts from 0

# 5. Save in multiple formats ------------------------------------------------------

# PDF format (vector graphics, suitable for publication)

ggsave(

filename = "GeneExpression_ViolinPlot.pdf",

plot = optimized_plot,

device = "pdf",

width = 12, # Width (inches)

height = 8, # Height (inches)

units = "in" # Unit

)

# PNG format (bitmap, high resolution)

ggsave(

filename = "GeneExpression_ViolinPlot.png",

plot = optimized_plot,

device = "png",

width = 12,

height = 8,

units = "in",

dpi = 600, # Resolution

bg = "white" # White background

)

# Display graphic in R

print(optimized_plot)

######## Combine Images ####

# Obtain death-related differential genes and draw Venn diagram ##

# Install necessary packages (if not already installed)

if (!require("VennDiagram")) install.packages("VennDiagram")

if (!require("gridExtra")) install.packages("gridExtra")

if (!require("dplyr")) install.packages("dplyr")

if (!require("ggplot2")) install.packages("ggplot2")

if (!require("tidyr")) install.packages("tidyr")

# Load necessary packages

library(VennDiagram) # For drawing Venn diagrams

library(dplyr) # For data processing

library(grid) # For graphic layout

library(gridExtra) # For combining graphics

library(ggplot2) # For drawing violin plots

library(tidyr) # For data tidying

### Combine all graphics ###

# Convert graphics to grob objects

venn_necro_grob <- gTree(children = venn_necro)

venn_pyro_grob <- gTree(children = venn_pyro)

venn_hub_grob <- gTree(children = venn_hub)

violin_grob <- ggplotGrob(optimized_plot)

# Create combined graphic layout

# First row: two Venn diagrams side by side

venn_row <- arrangeGrob(

venn_necro_grob,

venn_pyro_grob,

ncol = 2,

widths = c(1, 1) # Equal width

)

# Second row: hub gene Venn diagram and violin plot side by side

hub_violin_row <- arrangeGrob(

venn_hub_grob,

violin_grob,

ncol = 2,

widths = c(1, 2) # Violin plot width twice that of Venn diagram

)

# Combine all rows

combined_plot <- arrangeGrob(

venn_row,

hub_violin_row,

nrow = 2,

heights = c(1, 1.5), # Second row taller

top = textGrob("",

gp = gpar(fontsize = 16, fontface = "bold"))

)

### Add labels A, B, C, D, E ###

# Create function to add labels

add_label <- function(grob, label,

x = 0.02, y = 0.98,

size = 24, fontface = "bold") {

arrangeGrob(

grob,

top = textGrob(label,

x = unit(x, "npc"),

y = unit(y, "npc"),

hjust = 0,

gp = gpar(fontsize = size, fontface = fontface))

)

}

# Add labels to each subplot

venn_necro_labeled <- add_label(venn_necro_grob, "A")

venn_pyro_labeled <- add_label(venn_pyro_grob, "B")

venn_hub_labeled <- add_label(venn_hub_grob, "C")

violin_labeled <- add_label(violin_grob, "D")

# Recreate combined plot with labels

venn_row_labeled <- arrangeGrob(

venn_necro_labeled,

venn_pyro_labeled,

ncol = 2,

widths = c(1, 1)

)

hub_violin_row_labeled <- arrangeGrob(

venn_hub_labeled,

violin_labeled,

ncol = 2,

widths = c(1, 2)

)

combined_plot_labeled <- arrangeGrob(

venn_row_labeled,

hub_violin_row_labeled,

nrow = 2,

heights = c(1, 1.5),

top = textGrob("",

gp = gpar(fontsize = 16, fontface = "bold"))

)

# Add label E to entire combined plot

final_plot <- add_label(combined_plot_labeled, "", x = 0.01, y = 0.99)

### Save combined graphics ###

# Save as PDF

pdf("results/Combined_Figures.pdf", width = 14, height = 12)

grid.draw(final_plot)

dev.off()

# Save as PNG (high resolution)

png("results/Combined_Figures.png", width = 1400, height = 1200, res = 150)

grid.draw(final_plot)

dev.off()

# Save as TIFF format (high resolution 600 DPI)

tiff("results/Combined_Figures.tiff",

width = 14, # Width 14 inches

height = 12, # Height 12 inches

units = "in", # Unit: inches

res = 600, # Resolution 600 DPI

compression = "lzw") # LZW compression to reduce file size

grid.draw(final_plot)

dev.off()

# Print result summary

cat("Analysis completed! Results saved to results directory\n")

cat("Necroptosis intersection genes:", length(necroptosis_intersect), "\n")

cat("Pyroptosis intersection genes:", length(pyroptosis_intersect), "\n")

cat("Total death-related differential genes:", nrow(P_N_gene), "\n")

##### Step 5: Protein-Protein Interaction (PPI) Network and Screening Hub Genes via Cytohubba Plugin ##################

# Read differential gene expression matrix for necroptosis and pyroptosis intersection

N_P_expr <- read.csv("N_P_expression_Matrix.csv", row.names = 1)

# Obtain expression matrix of 6 genes retained after PPI analysis

PPI_expr <- N_P_expr[-7,]

# Save expression matrix

write.csv(PPI_expr, "./PPI/PPI_expression_Matrix.csv")

# Obtain intersection of MCC, MNC, Degree, EPC four algorithms

# Load necessary packages

library(VennDiagram) # For drawing Venn diagrams

library(grid) # Provides graphic output support

setwd("./PPI")

# Step 1: Read four CSV files and extract top 4 genes

mcc_genes <- read.csv("MCC.csv")$Name[1:5] # Extract top 5 gene names from MCC file

mnc_genes <- read.csv("MNC.csv")$Name[1:5] # Extract top 5 gene names from MNC file

degree_genes <- read.csv("Degree.csv")$Name[1:5] # Extract top 5 gene names from Degree file

epc_genes <- read.csv("EPC.csv")$Name[1:5] # Extract top 5 gene names from EPC file

# Step 2: Create gene list (named list for Venn diagram labels)

gene_list <- list(

MCC = mcc_genes,

MNC = mnc_genes,

Degree = degree_genes,

EPC = epc_genes

)

# Step 3: Calculate intersection genes

intersect_genes <- Reduce(intersect, gene_list) # Get intersection of four sets

# Save intersection genes

write.csv(intersect_genes, "Hub Gene.csv")

# Step 4: Print intersection genes (optional)

print(paste("Intersection genes:", paste(intersect_genes, collapse = ", ")))

# Step 5: Draw Venn diagram

venn.plot <- venn.diagram(

x = gene_list,

filename = NULL, # Do not save directly to file

output = TRUE,

# Set graphic parameters

height = 3000, # Graphic height (pixels)

width = 3000, # Graphic width (pixels)

resolution = 300, # Resolution (DPI)

# Set label style

cat.cex = 1.2, # Set name font size

cex = 1.5, # Number label font size

# Set colors (use different colors to distinguish sets)

fill = c("#F8766D", "#7CAE00", "#00BFC4", "#C77CFF"),

# Set title

main = "Hub Gene",

main.cex = 1.5,

# Set set names

category.names = names(gene_list)

)

# Step 6: Display Venn diagram

grid.newpage() # Create new graphic page

grid.draw(venn.plot) # Draw Venn diagram

# Step 7: Save graphic to file

png("VennDiagram.png", width = 10, height = 10, units = "in", res = 300)

grid.draw(venn.plot)

dev.off()

# Save PDF format

pdf("VennDiagram.pdf", width = 10, height = 10) # Width and height units in inches

grid.draw(venn.plot) # Draw previously created Venn diagram object

dev.off() # Close graphic device

# Obtain hub gene expression matrix and draw heatmap

library(tidyverse) # Data processing and visualization

library(corrplot) # Correlation heatmap drawing

library(ggpubr) # Statistical plotting

# Step 1: Data preparation and preprocessing

# Read expression matrix

expr_data <- read.csv("GSE_1.csv", row.names = 1)

print(paste("Expression matrix dimensions:", dim(expr_data)[1], "genes ×", dim(expr_data)[2], "samples"))

# Read hub genes

hub_genes <- read.csv("Hub Gene.csv")$Gene

print(paste("Hub genes:", paste(hub_genes, collapse = ", ")))

# Check presence of hub genes in expression matrix

missing_genes <- hub_genes[!hub_genes %in% rownames(expr_data)]

if (length(missing_genes) > 0) {

warning(paste("The following hub genes are not in expression matrix:", paste(missing_genes, collapse = ", ")))

}

# Extract hub gene expression data (only keep existing genes)

hub_expr <- expr_data[rownames(expr_data) %in% hub_genes, ]

print("Analyzable hub genes:")

print(rownames(hub_expr))

write.csv(hub_expr, "Hub_expr.csv")

# Draw hub gene heatmap

library(pheatmap) # For drawing heatmaps

library(RColorBrewer) # Provides color schemes

packageVersion("pheatmap")

# Step 1: Read data

# Note: First column is gene names, should be set as row names

data <- read.csv("Hub_expr.csv", row.names = 1, check.names = FALSE)

# Check data structure

str(data) # Confirm genes in rows, samples in columns

# Step 2: Data standardization (standardize by gene rows)

# Heatmaps usually need standardization to highlight expression patterns

scaled_data <- t(scale(t(data))) # Transpose then standardize by rows, then transpose back

# Step 3: Create sample grouping annotation (judge based on sample names)

# Create grouping based on sample numbers: first 4 are Group1, last 4 are Group2

sample_groups <- data.frame(

Group = rep(c("Control", "IRI"), each = 4)

)

rownames(sample_groups) <- colnames(data)

# Step 4: Set heatmap colors

heatmap_colors <- colorRampPalette(rev(brewer.pal(11, "RdBu")))(100)

# Step 5: Draw heatmap

pheatmap(

mat = scaled_data, # Standardized data

color = heatmap_colors, # Color mapping

border_color = NA, # No cell borders

show_rownames = TRUE, # Show row names (gene names)

show_colnames = TRUE, # Show column names (sample IDs)

annotation_col = sample_groups, # Add grouping annotation to columns

cluster_rows = TRUE, # Cluster genes

cluster_cols = FALSE, # Do not cluster samples (keep original order)

scale = "none", # Already manually standardized, no scaling here

fontsize_row = 10, # Row name font size

fontsize_col = 8, # Column name font size

main = "Hub Genes Expression Heatmap", # Title

annotation_names_col = TRUE # Show grouping annotation title

)

########## WGCNA Co-expression Network Analysis #######

setwd("./WGCNA")

library(WGCNA)

mydata<-read.csv("GSE_1.csv",row.names = 1)

datExpr0 = data.frame(t(mydata))

colnames(datExpr0) <- rownames(mydata)

rownames(datExpr0) <- colnames(mydata)

# Filter genes with top 25% variance

datExpr1<-datExpr0

m.vars=apply(datExpr0,2,var)

expro.upper=datExpr0[,which(m.vars>quantile(m.vars, probs = seq(0, 1, 0.25))[4])]

datExpr1<-data.matrix(expro.upper)

# Check for bad samples or genes

gsg = goodSamplesGenes(datExpr1, verbose = 3);

gsg$allOK

# If the returned result here is TRUE, it means all genes passed the check.

# If you use all genes as input, it's likely to return FALSE, indicating bad genes or samples exist.

# The following code will remove those bad genes or samples.

# Remove bad samples or genes

if (!gsg$allOK){

# Optionally, print the gene and sample names that were removed:

if (sum(!gsg$goodGenes)>0)

printFlush(paste("Removing genes:", paste(names(datExpr0)[!gsg$goodGenes], collapse = ", ")));

if (sum(!gsg$goodSamples)>0)

printFlush(paste("Removing samples:", paste(rownames(datExpr1)[!gsg$goodSamples], collapse = ", ")));

# Remove the offending genes and samples from the data:

datExpr1 = datExpr1[gsg$goodSamples, gsg$goodGenes]

}

# Check for outlier samples

sampleTree = hclust(dist(datExpr1), method = "average")

par(cex = 0.7);

par(mar = c(0,4,2,0))

plot(sampleTree, main = "Sample clustering to detect outliers", sub="", xlab="", cex.lab = 1.5,

cex.axis = 1.5, cex.main = 2)

# If your data has outlier samples that need removal, run the following code.

# Remove outlier samples

#plot(sampleTree, main = "Sample clustering to detect outliers", sub="", xlab="", cex.lab = 1.5, cex.axis = 1.5, cex.main = 2) +

# Where to cut, replace "h = 110" and "cutHeight = 110" with your cutoff

#abline(h = 150, col = "red")

#clust = cutreeStatic(sampleTree, cutHeight = 150, minSize = 10)

#keepSamples = (clust==1)

#datExpr = datExpr1[keepSamples, ]

#nGenes = ncol(datExpr)

#nSamples = nrow(datExpr)

#dim(datExpr)

#datExpr<-as.data.frame(datExpr)

# If no need to remove outlier samples, run the following code

datExpr = as.data.frame(datExpr1)

nGenes = ncol(datExpr)

nSamples = nrow(datExpr)

# Choose appropriate threshold for network construction

# Through this step calculation, find the minimum power (soft threshold) where scale free topology model fit approaches 0.9, for next step network construction.

powers = c(c(1:10), seq(from = 12, to=20, by=2))

sft = pickSoftThreshold(datExpr, powerVector = powers, verbose = 5)

pdf("1Threshold.pdf",width = 10, height = 5)

par(mfrow = c(1,2))

cex1 = 0.9

plot(sft$fitIndices[,1], -sign(sft$fitIndices[,3])*sft$fitIndices[,2],

xlab="Soft Threshold (power)",ylab="Scale Free Topology Model Fit,signed R^2",type="n",

main = paste("Scale independence")) +

text(sft$fitIndices[,1], -sign(sft$fitIndices[,3])*sft$fitIndices[,2],

labels=powers,cex=cex1,col="red")+

abline(h=0.90,col="red")

plot(sft$fitIndices[,1], sft$fitIndices[,5],

xlab="Soft Threshold (power)",ylab="Mean Connectivity", type="n",

main = paste("Mean connectivity")) +

text(sft$fitIndices[,1], sft$fitIndices[,5], labels=powers, cex=cex1,col="red")

dev.off()

# Construct network, find gene modules

net = blockwiseModules(datExpr, power = 14,

TOMType = "unsigned", minModuleSize = 30,

reassignThreshold = 0, mergeCutHeight = 0.25,

numericLabels = TRUE, pamRespectsDendro = FALSE,

saveTOMs = TRUE,

#saveTOMFileBase = "MyTOM",

verbose = 3)

table(net$colors)

mergedColors = labels2colors(net$colors)

pdf("2module.pdf",width = 10, height = 5)

plotDendroAndColors(net$dendrograms[[1]], mergedColors[net$blockGenes[[1]]], "Module colors",

dendroLabels = FALSE, hang = 0.03,

addGuide = TRUE, guideHang = 0.05)

dev.off()

moduleLabels = net$colors

moduleColors = labels2colors(net$colors)

MEs = net$MEs;

geneTree = net$dendrograms[[1]]

# Output gene modules to file

text <- unique(moduleColors)

for (i in 1:length(text)) {

y=t(assign(paste(text[i],"expr",sep = "."),datExpr[moduleColors==text[i]]))

write.csv(y,paste(text[i],"csv",sep = "."),quote = F)

}

# Phenotype and module correlation

samples_info <- read.csv('GSE_1_group.csv', row.names = 1)

# Ensure sample grouping data matches expression matrix sample order

samples_info <- samples_info[rownames(datExpr), , drop = FALSE]

# Create two dummy variables representing Control group and IRI group respectively

traitData <- data.frame(

Control = as.numeric(samples_info$group == "Control"),

IRI = as.numeric(samples_info$group == "IRI")

)

rownames(traitData) <- rownames(samples_info)

# Calculate module eigengenes

moduleLabelsAutomatic <- net$colors

moduleColorsAutomatic <- labels2colors(moduleLabelsAutomatic)

MEs0 <- moduleEigengenes(datExpr, moduleColorsAutomatic)$eigengenes

MEs <- orderMEs(MEs0)

# Calculate correlation between modules and two groupings

modTraitCor <- cor(MEs, traitData, use = "p")

modTraitP <- corPvalueStudent(modTraitCor, nSamples)

# Prepare heatmap text matrix

textMatrix <- paste(signif(modTraitCor, 2), "\n(", signif(modTraitP, 1), ")", sep = "")

dim(textMatrix) <- dim(modTraitCor)

# Draw module-phenotype relationship heatmap

pdf("3Module-trait.pdf", width = 8, height = 8)

par(mar = c(6, 8.5, 3, 3))

labeledHeatmap(Matrix = modTraitCor,

xLabels = colnames(traitData),

yLabels = names(MEs),

ySymbols = names(MEs),

colorLabels = FALSE,

colors = blueWhiteRed(50),

textMatrix = textMatrix,

setStdMargins = FALSE,

cex.text = 0.7,

cex.lab = 0.8,

yColorWidth = 0.02,

xColorWidth = 0.05,

zlim = c(-1,1),

main = "Module-trait relationships (Separated by Group)")

dev.off()

# Output correlation results to file

cor_results <- data.frame(

Module = rep(names(MEs), 2),

Group = rep(colnames(traitData), each = nrow(modTraitCor)),

Correlation = c(modTraitCor[,1], modTraitCor[,2]),

Pvalue = c(modTraitP[,1], modTraitP[,2])

)

write.csv(cor_results, "module_trait_correlation_separated.csv", row.names = FALSE)

# Optional: Output top correlated modules for each group separately

control_cor <- data.frame(

Module = names(MEs),

Correlation = modTraitCor[,1],

Pvalue = modTraitP[,1]

)

control_cor <- control_cor[order(-abs(control_cor$Correlation)), ]

iri_cor <- data.frame(

Module = names(MEs),

Correlation = modTraitCor[,2],

Pvalue = modTraitP[,2]

)

iri_cor <- iri_cor[order(-abs(iri_cor$Correlation)), ]

write.csv(control_cor, "module_control_correlation.csv", row.names = FALSE)

write.csv(iri_cor, "module_IRI_correlation.csv", row.names = FALSE)

# Select most relevant module MEturquoise to validate hub genes

# Load necessary packages

library(VennDiagram) # For drawing Venn diagrams

library(readr) # For reading CSV files

library(grid) # For graphic output

library(ggplot2) # For alternative plotting schemes

# Step 1: Read data

# Read hub gene list

hub_genes <- read_csv("Hub Gene.csv")

# Read WGCNA module gene expression matrix

turquoise_module <- read_csv("turquoise.csv")

# Step 2: Data preprocessing and cleaning

# Extract hub gene names (convert to character vector)

hub_gene_list <- as.character(hub_genes$Gene)

# Extract gene names from WGCNA module

# First column of turquoise module data is gene names

turquoise_gene_list <- as.character(turquoise_module[[1]])

# Check basic data information

cat("Number of hub genes:", length(hub_gene_list), "\n")

cat("Number of WGCNA module genes:", length(turquoise_gene_list), "\n")

# Step 3: Calculate intersection

# Find genes present in both hub genes and WGCNA module

common_genes <- intersect(hub_gene_list, turquoise_gene_list)

# Output intersection results

cat("Number of common genes:", length(common_genes), "\n")

cat("Common genes:", paste(common_genes, collapse = ", "), "\n")

# Create output directory (if not exists)

if (!dir.exists("results")) {

dir.create("results")

}

# Option 1: Adjust Venn diagram parameters to make small sets more obvious

pdf("results/Venn_Diagram_Adjusted.pdf", width = 10, height = 8)

# Create custom Venn diagram, adjust proportions to make small sets more obvious

venn_plot <- draw.pairwise.venn(

area1 = length(hub_gene_list), # Size of set 1

area2 = length(turquoise_gene_list), # Size of set 2

cross.area = length(common_genes), # Intersection size

category = c("Hub Genes", "Turquoise Module"), # Set labels

fill = c("#FF6B6B", "#4ECDC4"), # More vivid colors

alpha = 0.7, # Increase transparency

lty = "solid", # Solid border

lwd = 3, # Increase border width

cex = 2, # Increase number size

cat.cex = 1.8, # Increase category label size

cat.pos = c(-20, 20), # Adjust category label position

cat.dist = 0.08, # Adjust category label distance

cat.just = list(c(0.5, 1), c(0.5, 1)), # Center alignment

ext.pos = 0, # Intersection label position

ext.dist = -0.05, # Intersection label distance

ext.length = 0.9, # External line length

ext.line.lwd = 2, # External line width

rotation.degree = 0, # Rotation angle

scaled = FALSE # Do not scale proportionally, make small sets more obvious

)

# Add title and description

grid.text("Venn Diagram: Hub Genes vs Turquoise Module",

x = 0.5, y = 0.95,

gp = gpar(fontsize = 18, fontface = "bold"))

# Add detailed description text

grid.text(paste("Hub Genes (n=", length(hub_gene_list), "): ", paste(hub_gene_list, collapse = ", ")),

x = 0.05, y = 0.05, just = "left",

gp = gpar(fontsize = 10, col = "darkred"))

grid.text(paste("Common Genes (n=", length(common_genes), "): ", paste(common_genes, collapse = ", ")),

x = 0.05, y = 0.02, just = "left",

gp = gpar(fontsize = 10, col = "darkblue", fontface = "bold"))

dev.off()

####### Step 6: Validate Hub Gene Expression Through Internal Validation Set and Independent Validation Set #######

# Load necessary packages

library(tidyverse) # Data processing and plotting

library(ggpubr) # Add statistical test results

# Step 1: Read data

# Read expression matrix file (validation set)

expr_data <- read.csv("GSE_2.csv", row.names = 1, check.names = FALSE)

# Read hub gene list

hub_genes <- read.csv("Hub Gene.csv")$Gene

head(hub_genes)

# Step 2: Prepare data

# Transpose data frame so genes are columns, samples are rows

expr_data_t <- as.data.frame(t(expr_data))

# Add grouping information (first 4 samples are Control, last 4 are IRI)

expr_data_t$Group <- rep(c("Control", "IRI"), each = 4)

# Step 3: Filter hub gene data

# Check if hub genes exist in data

available_genes <- hub_genes[hub_genes %in% colnames(expr_data_t)]

missing_genes <- setdiff(hub_genes, available_genes)

if (length(missing_genes) > 0) {

message("The following hub genes are missing from expression matrix: ", paste(missing_genes, collapse = ", "))

}

# Extract hub gene data (including grouping information)

hub_expr <- expr_data_t[, c(available_genes, "Group")]

# Save internal validation set hub gene expression matrix

hub_expr_1 <- as.data.frame(t(hub_expr))

Hub_expr_test <- hub_expr_1[-5,]

write.csv(Hub_expr_test, "Hub_expr_test.csv")

# Step 4: Convert data format (wide to long format)

hub_expr_long <- hub_expr %>%

pivot_longer(

cols = -Group,

names_to = "Gene",

values_to = "Expression"

) %>%

mutate(

Group = factor(Group, levels = c("Control", "IRI")),

Gene = factor(Gene, levels = available_genes)

)

# Step 5: Draw violin plot

violin_plot <- ggplot(hub_expr_long, aes(x = Group, y = Expression, fill = Group)) +

geom_violin(trim = FALSE, alpha = 0.7) +

geom_boxplot(width = 0.1, fill = "white", outlier.shape = NA) +

geom_jitter(size = 2, width = 0.1, height = 0, alpha = 0.7) +

facet_wrap(~ Gene, scales = "free_y", ncol = 2) +

stat_compare_means(

method = "wilcox.test", # Use Wilcoxon test (non-parametric method, suitable for small samples)

label = "p.format", # Display p-value

label.x = 1.5, # p-value position

size = 5,

show.legend = FALSE

) +

labs(

title = "Expression of Hub Genes in Internal Validation Set",

x = "Group",

y = "Expression Level (log2)",

caption = "First 4 samples: Control, Last 4 samples: IRI"

) +

scale_fill_manual(values = c("Control" = "blue", "IRI" = "red")) +

theme_bw(base_size = 14) +

theme(

plot.title = element_text(hjust = 0.5, face = "bold"),

legend.position = "top",

strip.text = element_text(face = "bold"),

strip.background = element_rect(fill = "gray90")

)

# Step 6: Save images

# Save PDF

pdf_plot <- ggsave(

filename = "Validation_Violin_Plots.pdf",

plot = violin_plot,

width = 10, # Image width (inches)

height = 11, # Image height (inches)

dpi = 600 # Resolution

)

# Save TIFF (add tiff format output)

tiff_plot <- ggsave(

filename = "Validation_Violin_Plots.tiff",

plot = violin_plot,

width = 10, # Keep same dimensions as PDF

height = 8,

units = "in", # Unit set to inches

dpi = 300, # High resolution 300dpi

compression = "lzw" # Use LZW lossless compression

)

# Step 7: Display results

print(paste("Successfully generated violin plot containing", length(available_genes), "hub genes"))

print(paste("PDF image saved as: Validation_Violin_Plots.pdf"))

print(paste("TIFF image saved as: Validation_Violin_Plots.tiff (300 dpi)"))

######### Independent Validation Set Validation of Hub Genes

setwd("./GSE232246")

# Load necessary packages

library(ggplot2)

library(ggpubr)

library(dplyr)

library(tidyr)

# Read data

fpkm_data <- read.csv("GSE232246_FPKM_gene_avg.csv", row.names = 1)

hub_genes <- read.csv("Hub Gene.csv")$Gene

# Check if hub genes are in dataset

available_genes <- hub_genes[hub_genes %in% rownames(fpkm_data)]

print(paste("Available hub genes:", paste(available_genes, collapse = ", ")))

# Extract hub gene expression data

hub_expr <- fpkm_data[available_genes, ]

# Ensure all expression data is numeric

hub_expr <- as.data.frame(apply(hub_expr, 2, as.numeric))

rownames(hub_expr) <- available_genes

# Create grouping information - use IRI instead of Ischemia

groups <- c(rep("IRI", 3), rep("Control", 3))

# Improved statistical test function

perform_multiple_tests <- function(gene_expr, groups, gene_name) {

# Ensure data is numeric

expr_values <- as.numeric(gene_expr)

# Check if there are enough non-missing observations

if(sum(!is.na(expr_values[groups == "IRI"])) < 2 ||

sum(!is.na(expr_values[groups == "Control"])) < 2) {

warning(paste("Insufficient data for statistical test, gene:", gene_name))

return(data.frame(

Gene = gene_name,

t_test_p = NA,

wilcox_p = NA,

cohens_d = NA,

fold_change = NA,

mean_IRI = mean(expr_values[groups == "IRI"], na.rm = TRUE),

mean_control = mean(expr_values[groups == "Control"], na.rm = TRUE)

))

}

IRI <- expr_values[groups == "IRI"]

control <- expr_values[groups == "Control"]

# t-test

t_test <- tryCatch(

t.test(IRI, control, na.action = na.omit),

error = function(e) {

warning(paste("t-test failed, gene:", gene_name, "Error:", e$message))

return(list(p.value = NA))

}

)

# Wilcoxon test

wilcox_test <- tryCatch(

wilcox.test(IRI, control, na.action = na.omit),

error = function(e) {

warning(paste("Wilcoxon test failed, gene:", gene_name, "Error:", e$message))

return(list(p.value = NA))

}

)

# Effect size calculation (Cohen's d)

mean_diff <- mean(IRI, na.rm = TRUE) - mean(control, na.rm = TRUE)

pooled_sd <- sqrt(((length(na.omit(IRI))-1)*var(IRI, na.rm = TRUE) +

(length(na.omit(control))-1)*var(control, na.rm = TRUE)) /

(length(na.omit(IRI)) + length(na.omit(control)) - 2))

cohens_d <- ifelse(pooled_sd > 0, mean_diff / pooled_sd, NA)

# Fold change

fold_change <- ifelse(mean(control, na.rm = TRUE) > 0,

mean(IRI, na.rm = TRUE) / mean(control, na.rm = TRUE),

NA)

return(data.frame(

Gene = gene_name,

t_test_p = ifelse(is.null(t_test$p.value), NA, t_test$p.value),

wilcox_p = ifelse(is.null(wilcox_test$p.value), NA, wilcox_test$p.value),

cohens_d = cohens_d,

fold_change = fold_change,

mean_IRI = mean(IRI, na.rm = TRUE),

mean_control = mean(control, na.rm = TRUE)

))

}

# Perform statistical tests

results_list <- list()

for(i in 1:nrow(hub_expr)) {

gene_name <- rownames(hub_expr)[i]

results_list[[i]] <- perform_multiple_tests(hub_expr[i, ], groups, gene_name)

}

results <- do.call(rbind, results_list)

# FDR correction (only for valid p-values)

valid_t_p <- !is.na(results$t_test_p)

valid_wilcox_p <- !is.na(results$wilcox_p)

results$t_test_fdr <- NA

results$wilcox_fdr <- NA

if(sum(valid_t_p) > 0) {

results$t_test_fdr[valid_t_p] <- p.adjust(results$t_test_p[valid_t_p], method = "fdr")

}

if(sum(valid_wilcox_p) > 0) {

results$wilcox_fdr[valid_wilcox_p] <- p.adjust(results$wilcox_p[valid_wilcox_p], method = "fdr")

}

print(results)

# Visualization function

plot_gene_expression <- function(gene_name) {

gene_data <- data.frame(

Expression = as.numeric(hub_expr[gene_name, ]),

Group = groups

)

p_value <- results[results$Gene == gene_name, "wilcox_p"]

fc <- results[results$Gene == gene_name, "fold_change"]

# Create title

if(is.na(p_value) || is.na(fc)) {

plot_title <- paste(gene_name, "\nInsufficient data for statistical test")

} else {

plot_title <- paste(gene_name, "\nWilcoxon p =", round(p_value, 4),

"FC =", round(fc, 2))

}

ggplot(gene_data, aes(x = Group, y = Expression, fill = Group)) +

geom_boxplot(alpha = 0.7) +

geom_point(size = 3, position = position_jitter(width = 0.2)) +

labs(title = plot_title, y = "FPKM") +

theme_minimal() +

scale_fill_manual(values = c("Control" = "lightblue", "IRI" = "salmon")) +

theme(plot.title = element_text(size = 10, face = "bold"))

}

# Generate graphics for all hub genes

if(length(available_genes) > 0) {

plots <- lapply(available_genes, plot_gene_expression)

# Calculate appropriate graphic layout

n_genes <- length(available_genes)

n_col <- 2

n_row <- ceiling(n_genes / n_col)

# Combine graphics and add overall title

combined_plot <- ggarrange(plotlist = plots, ncol = n_col, nrow = n_row)

combined_plot <- annotate_figure(combined_plot,

top = text_grob("Independent validation set verifies hub genes",

face = "bold", size = 16))

# Save PDF file - use multiple methods to avoid write errors

pdf_file <- "Hub_Genes_Expression_Plot.pdf"

success <- FALSE

# Method 1: Use pdf() device

tryCatch({

pdf(pdf_file, width = 8, height = 4 * n_row + 0.5)

print(combined_plot)

dev.off()

print(paste("Visualization results saved as:", pdf_file))

success <- TRUE

}, error = function(e) {

print(paste("PDF device save failed:", e$message))

})

# Method 2: If method 1 fails, try using ggsave

if (!success) {

tryCatch({

ggsave(pdf_file,

plot = combined_plot,

device = "pdf",

width = 8,

height = 4 * n_row + 0.5,

dpi = 600,

units = "in")

print(paste("Visualization results saved as:", pdf_file))

success <- TRUE

}, error = function(e) {

print(paste("ggsave PDF save failed:", e$message))

})

}

# Method 3: If both previous methods fail, try saving as PNG

if (!success) {

png_file <- "Hub_Genes_Expression_Plot.png"

tryCatch({

ggsave(png_file,

plot = combined_plot,

device = "png",

width = 8,

height = 4 * n_row + 0.5,

dpi = 600,

units = "in")

print(paste("PDF save failed, visualization results saved as PNG:", png_file))

}, error = function(e) {

print(paste("PNG save also failed:", e$message))

})

}

# Also save statistical results

write.csv(results, "Hub_Genes_Statistical_Results.csv", row.names = FALSE)

print("Statistical results saved as: Hub_Genes_Statistical_Results.csv")

# Optional: Save each gene's graphic separately

dir.create("Individual_Gene_Plots", showWarnings = FALSE)

# Save individual graphic for each gene

for(i in seq_along(available_genes)) {

gene_name <- available_genes[i]

individual_plot <- plot_gene_expression(gene_name)

individual_pdf <- paste0("Individual_Gene_Plots/", gene_name, "_Expression.pdf")

success_individual <- FALSE

tryCatch({

ggsave(individual_pdf,

plot = individual_plot,

device = "pdf",

width = 6,

height = 6,

dpi = 600,

units = "in")

success_individual <- TRUE

}, error = function(e) {

print(paste("Single gene PDF save failed:", gene_name, e$message))

})

if (!success_individual) {

individual_png <- paste0("Individual_Gene_Plots/", gene_name, "_Expression.png")

tryCatch({

ggsave(individual_png,

plot = individual_plot,

device = "png",

width = 6,

height = 6,

dpi = 600,

units = "in")

print(paste("Gene", gene_name, "PDF save failed, saved as PNG"))

}, error = function(e) {

print(paste("Single gene PNG save also failed:", gene_name, e$message))

})

}

}

print(paste("Individual gene graphics saved to Individual_Gene_Plots directory"))

# Display graphic (optional) - add error handling

tryCatch({

print(combined_plot)

}, error = function(e) {

print("Unable to display graphic on screen, but file has been saved")

})

} else {

print("No available hub genes for visualization")

}

# Print session information for debugging

print("Analysis completed!")

print(sessionInfo())

########### Step 7: GO and KEGG Functional Enrichment ############

setwd("./GO和KEGG富集分析")

##### 1. GO Enrichment Analysis #######

BiocManager::install(c("clusterProfiler", "org.Mm.eg.db", "enrichplot", "ggplot2"))

# Load required packages

library(clusterProfiler) # Core package for enrichment analysis

library(tidyverse)

library(org.Mm.eg.db) # Mouse gene annotation database

library(enrichplot) # Visualization of enrichment results

library(ggplot2) # Plotting tool

#install.packages("viridis")

library(viridis) # Provides more beautiful color schemes

# Step 1: Read hub gene data

Hub_Gene <- read.csv("Hub Gene.csv", header = TRUE, stringsAsFactors = FALSE)

gene_symbols <- Hub_Gene$Gene # Extract gene Symbol list

head(gene_symbols)

# Step 2: Gene ID conversion (Symbol to Entrez ID)

gene_entrez <- mapIds(

org.Mm.eg.db, # Mouse database

keys = gene_symbols, # Input gene Symbols

column = "ENTREZID", # Target ID type

keytype = "SYMBOL", # Input ID type

multiVals = "first" # Take first when multiple matches

)

head(gene_entrez)

# Remove genes not matched to Entrez ID

gene_entrez <- na.omit(gene_entrez)

# Step 3: GO enrichment analysis (simultaneously includes BP/CC/MF)

go_results <- enrichGO(

gene = gene_entrez, # Target gene list

OrgDb = org.Mm.eg.db, # Species database

keyType = "ENTREZID", # Input gene ID type

ont = "ALL", # Simultaneously analyze three ontologies

pAdjustMethod = "BH", # p-value adjustment method (Benjamini-Hochberg)

pvalueCutoff = 0.05, # p-value threshold

qvalueCutoff = 0.2, # q-value threshold

readable = TRUE # Convert Entrez ID to readable gene Symbol

)

# Step 4: Result visualization (improved graphic size and color)

# 4.1 Bar plot (sorted by count) - improved version

if (nrow(go_results) > 0) {

# Create custom color mapping

ontology_colors <- c(BP = "#1f77b4", CC = "#ff7f0e", MF = "#2ca02c")

bar_plot <- barplot(go_results,

showCategory = 5, # Display top 5 entries

split = "ONTOLOGY", # Group by ontology type

font.size = 10, # Increase font size

color = "p.adjust") + # Color by adjusted p-value

scale_fill_viridis_c(option = "C", direction = -1) + # Use viridis color scheme

facet_grid(ONTOLOGY~., scale = "free") +

ggtitle("GO Enrichment Analysis") +

theme_minimal(base_size = 12) + # Use minimal theme

theme(legend.position = "right",

plot.title = element_text(face = "bold", size = 14),

strip.text = element_text(face = "bold", size = 11),

strip.background = element_rect(fill = "grey90"),

axis.text.y = element_text(size = 7), # Reduce Y-axis label font size

axis.title.y = element_text(size = 10)) + # Maintain Y-axis title size

labs(fill = "Adjusted p-value")

# Save bar plot

ggsave("GO_barplot.pdf", bar_plot, width = 12, height = 8, dpi = 300)

print(bar_plot)

} else {

message("No significant GO enrichment results, skip bar plot drawing")

}

# 4.2 Dot plot (sorted by enrichment factor) - improved version

if (nrow(go_results) > 0) {

dot_plot <- dotplot(go_results,

showCategory = 5,

split = "ONTOLOGY",

font.size = 10,

color = "p.adjust") + # Color by adjusted p-value

scale_color_viridis_c(option = "B", direction = -1, end = 0.9) + # Use viridis color scheme

facet_grid(ONTOLOGY~., scale = "free") +

ggtitle("GO Enrichment Dotplot") +

theme_minimal(base_size = 12) + # Use minimal theme

theme(legend.position = "right",

plot.title = element_text(face = "bold", size = 14),

strip.text = element_text(face = "bold", size = 11),

strip.background = element_rect(fill = "grey90"),

axis.text.y = element_text(size = 7), # Reduce Y-axis label font size

axis.title.y = element_text(size = 10)) + # Maintain Y-axis title size

labs(color = "Adjusted p-value", size = "Gene count")

# Save dot plot

ggsave("GO_dotplot.png", dot_plot, width = 12, height = 8, dpi = 300)

print(dot_plot)

} else {

message("No significant GO enrichment results, skip dot plot drawing")

}

# Step 4: Save results

write.csv(as.data.frame(go_results), "GO_enrichment_results.csv", row.names = FALSE)

# Step 5: Result interpretation instructions

cat(

"Enrichment result explanation:\n",

"1. ONTOLOGY: BP=Biological Process, CC=Cellular Component, MF=Molecular Function\n",

"2. GeneRatio: Number of genes enriched in pathway / Total input genes\n",

"3. BgRatio: Number of genes in pathway in background / Total background genes\n",

"4. pvalue/p.adjust: Enrichment significance (smaller p-value more significant)\n",

"5. qvalue: p-value after false discovery rate correction\n\n",

"Visualization improvement explanation:\n",

"1. Use viridis color scheme to improve color contrast and readability\n",

"2. Increase font and graphic size to make labels clearer\n",

"3. Reduce Y-axis label font size in bar and dot plots (8pt)\n",

"4. All graphics saved as PNG files (300dpi)\n\n",

"Tip: View saved CSV file for complete results"

)

######### 2. KEGG Enrichment Analysis ########

# Load packages

library(clusterProfiler) # Core package for enrichment analysis

library(org.Mm.eg.db) # Mouse gene annotation database

library(enrichplot) # Visualization of enrichment results

library(ggplot2) # Plotting tool

library(viridis) # Provides more beautiful color schemes

#BiocManager::install("pathview")

library(pathview) # KEGG pathway visualization

# Step 1: Read hub gene data

Hub_Gene <- read.csv("Hub Gene.csv", header = TRUE, stringsAsFactors = FALSE)

gene_symbols <- Hub_Gene$Gene # Extract gene Symbol list

head(gene_symbols)

cat("Number of input genes:", length(gene_symbols), "\n")

# Step 2: Gene ID conversion (Symbol to Entrez ID)

gene_entrez <- mapIds(

org.Mm.eg.db, # Mouse database

keys = gene_symbols, # Input gene Symbols

column = "ENTREZID", # Target ID type

keytype = "SYMBOL", # Input ID type

multiVals = "first" # Take first when multiple matches

)

head(gene_entrez)

# Remove genes not matched to Entrez ID

gene_entrez <- na.omit(gene_entrez)

cat("Number of successfully matched genes:", length(gene_entrez), "\n")

# Step 3: KEGG enrichment analysis

kegg_results <- enrichKEGG(

gene = gene_entrez, # Target gene list

organism = "mmu", # Mouse KEGG code

keyType = "kegg", # Use KEGG ID

pvalueCutoff = 0.05, # p-value threshold

pAdjustMethod = "BH", # p-value adjustment method

qvalueCutoff = 0.2, # q-value threshold

minGSSize = 5, # Minimum gene set size

maxGSSize = 500, # Maximum gene set size

use_internal_data = FALSE # Use online KEGG database

)

# Check enrichment results

if (is.null(kegg_results) || nrow(kegg_results) == 0) {

stop("No significant KEGG enrichment results, please adjust pvalueCutoff or qvalueCutoff parameters")

}

# Step 4: Result visualization (optimize font size and color contrast)

# 4.1 Bar plot (sorted by count) - optimized version

bar_plot <- barplot(kegg_results,

showCategory = 15, # Display top 15 entries

font.size = 10, # Increase font size

color = "p.adjust") + # Color by adjusted p-value

scale_fill_viridis_c(option = "D", direction = -1, end = 0.9) + # Use viridis color scheme

ggtitle("KEGG Pathway Enrichment Analysis") +

theme_minimal(base_size = 12) + # Use minimal theme

theme(legend.position = "right",

plot.title = element_text(face = "bold", size = 14),

axis.text.y = element_text(size = 9), # Optimize Y-axis label size

axis.title = element_text(size = 11)) +

labs(fill = "Adjusted p-value", x = "Gene Count", y = "KEGG Pathway")

# Save bar plot

ggsave("KEGG_barplot.pdf", bar_plot, width = 10, height = 8, dpi = 300)

print(bar_plot)

# 4.2 Dot plot (sorted by enrichment factor) - optimized version

dot_plot <- dotplot(kegg_results,

showCategory = 15,

font.size = 10,

color = "p.adjust") + # Color by adjusted p-value

scale_color_viridis_c(option = "B", direction = -1, end = 0.9) + # Use viridis color scheme

ggtitle("KEGG Pathway Enrichment") +

theme_minimal(base_size = 12) + # Use minimal theme

theme(legend.position = "right",

plot.title = element_text(face = "bold", size = 14),

axis.text.y = element_text(size = 9), # Optimize Y-axis label size

axis.title = element_text(size = 11)) +

labs(color = "Adjusted p-value", size = "Gene Count",

x = "Gene Ratio", y = "KEGG Pathway")

# Save dot plot

ggsave("KEGG_dotplot.png", dot_plot, width = 10, height = 8, dpi = 300)

print(dot_plot)

# Step 5: Save results

write.csv(as.data.frame(kegg_results), "KEGG_enrichment_results.csv", row.names = FALSE)

# Step 6: Result interpretation instructions

cat(

"\nKEGG enrichment analysis completed! Result explanation:\n",

"1. Results saved to: KEGG_enrichment_results.csv\n",

"2. Visualization files:\n",

" - KEGG_barplot.png: Enriched pathway bar plot\n",

" - KEGG_dotplot.png: Enriched pathway dot plot\n",

" - KEGG_emapplot.png: Pathway relationship network plot\n",

" - Top_KEGG_Pathway_*.png: Most significant pathway plots\n",

"3. Font optimization:\n",

" - Axis labels: 9-11pt\n",

" - Title: 14pt bold\n",

" - Legend: 10pt\n",

"4. Color optimization:\n",

" - Use viridis high contrast color scheme\n",

" - Bar plot: purple to yellow gradient\n",

" - Dot plot: blue to purple gradient\n",

" - Pathway plot: red-gray-blue three colors\n",

"5. Suggest focusing on cell death-related pathways, such as:\n",

" - Apoptosis pathway\n",

" - Necroptosis pathway\n",

" - NF-kappa B signaling pathway\n",

" - TNF signaling pathway\n",

" - Inflammatory pathways\n"

)

##### 3. Combine GO and KEGG Enrichment Analysis Bubble Plots #####

library(cowplot)

# 1. Prepare GO enrichment dot plot

# Optimize dot plot display settings, ensure consistent style between two plots

if (exists("go_results") && nrow(go_results) > 0) {

# Use larger font and consistent color scheme

go_dot <- dotplot(go_results,

showCategory = 5, # Display 5 entries to ensure balanced combined plot

split = "ONTOLOGY",

font.size = 9,

color = "p.adjust") +

scale_color_viridis_c(

name = "Adjusted p-value", # Unified legend name

option = "C", # Use golden gradient scheme

direction = -1,

begin = 0.1, # Avoid using too light colors

end = 0.8) +

facet_grid(ONTOLOGY~., scale = "free") +

labs(title = "GO Enrichment") + # Use subtitle for easy combination

theme_minimal(base_size = 10) +

theme(

legend.position = "none", # Remove individual legend, use shared legend

plot.title = element_text(

face = "bold",

hjust = 0.5, # Center title

size = 11),

axis.text.y = element_text(

size = 8, # Optimize Y-axis label size

lineheight = 0.8),

strip.text = element_text(

face = "bold",

size = 9),

panel.grid.major = element_line(

color = "grey90",

linewidth = 0.25)) # Thinner grid lines

} else {

# Create empty plot placeholder in case of no results

go_dot <- ggplot() +

annotate("text", x=0, y=0, label="No significant GO results") +

theme_void()

}

# 2. Prepare KEGG enrichment dot plot

# Maintain same visual style as GO

kegg_dot <- dotplot(kegg_results,

showCategory = 10, # Display 10 entries to maintain balance

font.size = 9,

color = "p.adjust") +

scale_color_viridis_c(

name = "Adjusted p-value", # Unified legend name

option = "C", # Same color scheme as GO

direction = -1,

begin = 0.1,

end = 0.8) +

labs(title = "KEGG Pathway Enrichment") + # Use subtitle

theme_minimal(base_size = 10) +

theme(

legend.position = "none", # Remove individual legend

plot.title = element_text(

face = "bold",

hjust = 0.5,

size = 11),

axis.text.y = element_text(

size = 8, # Match GO label dimensions

lineheight = 0.8),

panel.grid.major = element_line(

color = "grey90",

linewidth = 0.25))

# 3. Extract shared legend

# Use smaller size to ensure it doesn't squeeze main plot

shared_legend <- get_legend(

go_dot +

guides(color = guide_colorbar(barwidth = 1.5, barheight = 5)) +

theme(

legend.position = "right",

legend.text = element_text(size = 8),

legend.title = element_text(size = 9))

)

# 4. Combine graphic layout

# Define professional academic combination layout

combo_plot <- plot_grid(

go_dot, # Left GO plot

kegg_dot, # Right KEGG plot

ncol = 2, # Two-column layout

rel_widths = c(1.3, 1), # GO width greater than KEGG (due to more facets)

labels = c("A", "B"), # Add academic markers

label_size = 12, # Label font size

axis = "tb", # Top and bottom align axes

align = "h" # Horizontal alignment

)

# 5. Add shared legend and main title

final_plot <- plot_grid(

combo_plot, # Main graphic combination

shared_legend, # Shared legend

rel_widths = c(5, 0.8), # Legend to main plot width ratio

nrow = 1 # Single row layout

) %>%

ggdraw() + # Convert to drawable object

draw_label(

" ",

fontface = "bold",

size = 14,

y = 0.99, # Place at top

x = 0.5, # Horizontal center

hjust = 0.5

)

# 6. Save high-resolution graphics

ggsave("Combined_Enrichment.pdf",

plot = final_plot,

width = 13, # Increase width to accommodate dual plot layout

height = 8, # Height suitable for displaying 10-15 entries

dpi = 600, # Print-level resolution

device = cairo_pdf) # Use cairo engine for fidelity

ggsave("Combined_Enrichment.png",

plot = final_plot,

width = 13,

height = 8,

dpi = 600,

bg = "white") # Set transparent background

# 7. Important optimization annotation instructions

cat(

"\nCombined plot optimization points:\n",

"1. Visual consistency:\n",

" - Same color scheme (viridis C)\n",

" - Matching fonts (axis labels 8pt, titles 11pt)\n",

" - Consistent grid line style (grey90, 0.25pt)\n",

"2. Layout optimization:\n",

" - GO:KEGG width ratio=1.3:1 (compensate for GO facet space)\n",

" - Shared vertical color legend (avoid redundancy)\n",

" - Main title centered at top (14pt bold)\n",

"3. Academic elements:\n",

" - Add A/B panel markers\n",

" - 600dpi print-level resolution\n",

" - PDF uses cairo engine (faithful vector graphics)\n",

"4. Size balance:\n",

" - GO displays 8 entries (including 3 ontologies)\n",

" - KEGG displays 10 pathways\n",

"5. File output:\n",

" - Combined_Enrichment.pdf (vector graphics)\n",

" - Combined_Enrichment.png (bitmap)\n\n",

"Tip: Combined plot width 13 inches suitable for academic paper double-column typesetting"

)

######## Step 8: Immune Infiltration Analysis, Revealing Connection Between Necroptosis/Pyroptosis and Immune Microenvironment, Explaining Mechanism ##########

# Set working directory

setwd("./Cibersort")

# Load necessary packages

library(tidyverse) # Data processing and visualization

library(corrplot) # Correlation heatmap drawing

library(ggpubr) # Statistical plotting

library(CIBERSORT) # Immune infiltration analysis (need to download CIBERSORT.R in advance)

# Step 1: Data preparation and preprocessing

# Read expression matrix

expr_data <- read.csv("GSE_1.csv", row.names = 1)

print(paste("Expression matrix dimensions:", dim(expr_data)[1], "genes ×", dim(expr_data)[2], "samples"))

# Read hub genes

hub_genes <- read.csv("Hub Gene.csv")$Gene

print(paste("Hub genes:", paste(hub_genes, collapse = ", ")))

# Check presence of hub genes in expression matrix

missing_genes <- hub_genes[!hub_genes %in% rownames(expr_data)]

if (length(missing_genes) > 0) {

warning(paste("The following hub genes are not in expression matrix:", paste(missing_genes, collapse = ", ")))

}

# Extract hub gene expression data (only keep existing genes)

hub_expr <- expr_data[rownames(expr_data) %in% hub_genes, ]

print("Analyzable hub genes:")

print(rownames(hub_expr))

write.csv(hub_expr, "Hub_expr.csv")

# Step 2: Immune infiltration analysis (CIBERSORT)

# Convert expression data to matrix format

expr_matrix <- as.matrix(expr_data)

# Run CIBERSORT

cibersort_results <- cibersort(

sig_matrix = "Mouse.txt", # Immune cell signature matrix

mixture_file = expr_matrix, # Use matrix format expression data

perm = 100, # Number of permutations

QN = TRUE # Quantile normalization

)

# Save immune infiltration results

write.csv(cibersort_results, "CIBERSORT_Results.csv")

#cibersort_results <- read.csv("CIBERSORT_Results.csv", row.names = 1)

# Step 3: Correlation analysis (Spearman)

# Remove 4 cells with zero values, retain 21 immune cells

cibersort_results <- cibersort_results[,1:25]# Only take 25 immune cell types

cibersort_results <- cibersort_results[,-c(4,9,13,16)]

write.csv(cibersort_results, "CIBERSORT-Results_1.csv")

# Combine hub gene expression with immune cell abundance

combined_data <- cbind(t(hub_expr), cibersort_results) # Only take 21 immune cell types

# Calculate Spearman correlation coefficient

cor_matrix <- cor(combined_data, method = "spearman", use = "complete.obs")

# Extract correlation submatrix for hub genes and immune cells

hub_cor <- cor_matrix[rownames(hub_expr), colnames(cibersort_results)[1:21]]

write.csv(hub_cor, "hub_cor.csv")

# Step 4: Visualize correlation results

# Create heatmap save function with title

save_corr_heatmap <- function(cor_mat, filename, plot_title = "") {

# Select save device based on file type

if (grepl("\\.pdf$", filename, ignore.case = TRUE)) {

cairo_pdf(

filename = filename,

width = 16,

height = 12,

family = "Arial"

)

} else {

png(filename, width = 1200, height = 900, res = 150) # Increase height to accommodate title

}

# Set graphic layout and margins

par(oma = c(0, 0, 2, 0)) # Increase top outer margin for title

# Create heatmap

corrplot(

cor_mat,

method = "color",

tl.col = "black",

tl.srt = 90,

addCoef.col = "black",

number.cex = ifelse(grepl("\\.pdf$", filename), 1.2, 0.7),

col = colorRampPalette(c("#6D9EC1", "white", "#E46726"))(100),

tl.cex = ifelse(grepl("\\.pdf$", filename), 1.6, 1.0),

cl.cex = ifelse(grepl("\\.pdf$", filename), 1.2, 1.0),

mar = c(0, 0, 0, 0) # Reduce internal heatmap margins

)

# Add title

title(main = plot_title, outer = TRUE, cex.main = 2.0, font.main = 2)

dev.off()

}

# Save heatmap with title

save_corr_heatmap(

hub_cor,

"HubGenes_ImmuneCells_Correlation.png",

plot_title = "Hub Genes - Immune Cells Correlation"

)

save_corr_heatmap(

hub_cor,

"HubGenes_ImmuneCells_Correlation.pdf",

plot_title = "Hub Genes - Immune Cells Correlation"

)

# Step 5: Key gene-cell pair visualization (dot plot)

generate_scatter <- function(gene, cell, data) {

ggscatter(

data = data.frame(

Expression = data[, gene],

Abundance = data[, cell]

),

x = "Expression",

y = "Abundance",

title = paste(gene, "vs", cell),

xlab = paste(gene, "Expression (log2)"),

ylab = paste(cell, "Abundance"),

add = "reg.line",

conf.int = TRUE,

cor.coef = TRUE,

cor.method = "spearman"

) + theme_minimal()

}

# Plot for each hub gene with significantly correlated immune cells

for (gene in rownames(hub_expr)) {

# Find top 3 immune cells most correlated with this gene

top_cells <- names(sort(abs(hub_cor[gene, ]), decreasing = TRUE))[1:3]

plots <- list()

for (i in seq_along(top_cells)) {

plots[[i]] <- generate_scatter(gene, top_cells[i], combined_data)

}

# Combine and save images

p <- ggarrange(plotlist = plots, ncol = 3)

ggsave(

filename = paste0(gene, "_correlation_plots.pdf"),

plot = p,

width = 15,

height = 5,

dpi = 300

)

}

# Step 6: Inter-group immune cell difference analysis

# Define sample grouping (first 4 Control, last 4 IRI)

group <- factor(c(rep("Control", 4), rep("IRI", 4)), levels = c("Control", "IRI"))

head(group)

# Create immune cell abundance data frame

immune_df <- as.data.frame(cibersort_results[, 1:21])

immune_df$Group <- group

# Draw inter-group immune cell difference box plot

immune_df_long <- immune_df %>%

pivot_longer(-Group, names_to = "CellType", values_to = "Abundance")

ggplot(immune_df_long, aes(x = CellType, y = Abundance, fill = Group)) +

geom_boxplot(outlier.shape = NA, width = 0.7) + # Adjust boxplot width

geom_point(

position = position_jitterdodge(

jitter.width = 0.15, # Reduce jitter width

dodge.width = 0.7 # Match boxplot width

),

size = 1.2, # Reduce point size

alpha = 0.6 # Increase transparency to avoid overlap

) +

labs(

title = "Immune Cell Abundance Between Groups",

x = "Immune Cell Types",

y = "Estimated Abundance"

) +

theme_minimal(base_size = 12) + # Set base font size

theme(

axis.text.x = element_text(

angle = 90, # Change to 45 degree angle for easier reading

hjust = 1, # Horizontal align to right

vjust = 1, # Vertical align to top

size = 12 # Appropriately reduce font size

),

legend.position = "top",

plot.title = element_text(hjust = 0.5, size = 14, face = "bold"), # Center title and bold

axis.title = element_text(size = 12), # Axis title size

panel.grid.major.x = element_blank(), # Remove vertical grid lines

plot.margin = margin(1, 1, 1, 1, "cm") # Increase margins

) +

scale_fill_manual(values = c("#1F77B4", "#FF7F0E")) + # Use more beautiful colors

scale_y_continuous(expand = expansion(mult = c(0.05, 0.1))) # Increase top space of y-axis

# Save graphic - adjust dimensions based on number of cell types

num_celltypes <- length(unique(immune_df_long$CellType))

plot_width <- min(12, max(8, num_celltypes * 0.5)) # Dynamically calculate width

plot_height <- 8 # Fixed height

ggsave("Inter-group Immune Cell Difference Box Plot.pdf",

width = plot_width,

height = plot_height,

dpi = 300)

# Save all results

save.image("Analysis_Results.RData")

print("Analysis completed! All results saved to working directory")

# Step 7

# Read and transpose immune cell data

immune <- read.csv("CIBERSORT-Results_1.csv", row.names=1)

immune[1:5,]

immune_matrix <- as.matrix(immune)

data_transposed <- t(immune_matrix)

# Draw bar chart

cellnum <- read.csv("CIBERSORT-Results_1.csv", row.names=1)

cell.prop <- apply(cellnum, 1, function(x){x/sum(x)})

my36colors <- c('#E5D2DD', '#53A85F', '#F1BB72', '#F3B1A0', '#D6E7A3', '#57C3F3',

'#476D87','#E95C59', '#E59CC4', '#AB3282', '#23452F', '#BD956A', '#8C549C',

'#585658','#9FA3A8', '#E0D4CA', '#5F3D69', '#C5DEBA', '#58A4C3', '#E4C755',

'#F7F398','#AA9A59', '#E63863', '#E39A35', '#C1E6F3', '#6778AE', '#91D0BE',

'#B53E2B', '#712820', '#DCC1DD', '#CCE0F5', '#CCC9E6', '#625D9E', '#68A180',

'#3A6963','#968175')

data4plot <- data.frame()

for (i in 1:ncol(cell.prop)) {

data4plot <- rbind(

data4plot,

cbind(cell.prop[,i],rownames(cell.prop),

rep(colnames(cell.prop)[i],nrow(cell.prop)

)

)

)

}

colnames(data4plot) <- c('proportion','celltype','sample')

data4plot$proportion <- as.numeric(data4plot$proportion)

pdf(file="Immune Infiltration 1.pdf",height=10,width=22)

ggplot(data4plot,aes(sample,proportion,fill=celltype))+

geom_bar(stat="identity",position="fill")+

scale_fill_manual(values=my36colors)+

ggtitle("Cell Composition")+

theme_bw()+

theme(

# Adjust font size

axis.text.x = element_text(angle = 45, hjust = 1, vjust = 1, size = 20), # X-axis labels

axis.text.y = element_text(size = 12), # Y-axis labels

axis.title.x = element_text(size = 14), # X-axis title

axis.title.y = element_text(size = 14), # Y-axis title

plot.title = element_text(size = 20, hjust = 0.5), # Main title

legend.text = element_text(size = 20), # Legend text

legend.title = element_text(size = 20), # Legend title

# Other adjustments

axis.ticks.length = unit(0.3, 'cm'),

panel.grid.major = element_blank(), # Remove major grid lines

panel.grid.minor = element_blank() # Remove minor grid lines

) +

#labs(x = "Sample", y = "Proportion") + # Add axis titles

guides(fill = guide_legend(title = "Cell Type")) # Add legend title

dev.off()

# Correlation plot

pdf("Immune Correlation Plot.pdf", height=13, width=13)

par(oma=c(0.5, 1, 1, 1.2))

immune <- immune[, colMeans(immune) > 0]

M <- cor(immune)

corrplot(M,

order="hclust",

method="color",

addCoef.col="black",

diag=TRUE,

tl.col="black",

col=colorRampPalette(c("blue", "white", "red"))(50))

dev.off()

###### Combine ########

# Load necessary packages

library(tidyverse)

library(corrplot)

library(ggpubr)

library(ggplot2)

library(patchwork)

library(grid)

library(gridExtra)

library(gtable)

library(cowplot)

library(gridGraphics)

# Step 1: Create three graphic objects without using temporary files

# 1.1 Immune infiltration stacked bar chart - create directly usable graphic object

create_composition_plot <- function() {

ggplot(data4plot, aes(sample, proportion, fill = celltype)) +

geom_bar(stat = "identity", position = "fill") +

scale_fill_manual(values = my36colors) +

labs(title = "Immune Cell Composition",

x = "Sample",

y = "Proportion",

fill = "Cell Type") +

theme_minimal() +

theme(

legend.position = "right",

axis.text.x = element_text(angle = 45, hjust = 1, vjust = 1, size = 7),

axis.text.y = element_text(size = 8),

plot.title = element_text(face = "bold", size = 10, hjust = 0.5),

legend.text = element_text(size = 6),

legend.title = element_text(size = 7),

panel.grid.major = element_blank(),

panel.grid.minor = element_blank()

)

}

p_composition <- create_composition_plot()

# 1.2 Inter-group immune cell difference box plot

p_box <- ggplot(immune_df_long, aes(x = CellType, y = Abundance, fill = Group)) +

geom_boxplot(outlier.shape = NA, width = 0.7) +

geom_point(position = position_jitterdodge(jitter.width = 0.15, dodge.width = 0.7),

size = 1, alpha = 0.6) +

labs(title = "Immune Cell Abundance Between Groups",

x = "Immune Cell Types",

y = "Estimated Abundance") +

scale_fill_manual(values = c("#1F77B4", "#FF7F0E")) +

theme_minimal() +

theme(

axis.text.x = element_text(angle = 90, hjust = 1, vjust = 0.5, size = 7),

plot.title = element_text(face = "bold", size = 10, hjust = 0.5),

legend.position = "bottom",

axis.title = element_text(size = 8),

panel.grid.major = element_blank()

)

# 1.3 Hub gene-immune cell correlation heatmap - convert to grid graphic

create_corr_grob <- function(cor_mat) {

# Create virtual device

dev.control(displaylist = "enable")

# Create heatmap

par(oma = c(0, 0, 2, 0))

corrplot(

cor_mat,

method = "color",

tl.col = "black",

tl.srt = 60, # Adjust label angle for easier reading

addCoef.col = "black",

number.cex = 0.6, # Reduce coefficient font

tl.cex = 0.7, # Reduce label font

cl.cex = 0.7, # Reduce legend font

mar = c(0, 0, 0, 0),

col = colorRampPalette(c("#6D9EC1", "white", "#E46726"))(100)

)

title(main = "Hub Genes - Immune Cells Correlation",

outer = TRUE,

cex.main = 1.2,

font.main = 2)

# Capture graphic

grid.echo()

grid.grab()

}

corr_grob <- create_corr_grob(hub_cor)

# Step 2: Optimize layout and combine charts

# 2.1 Create combined layout

layout <- "

A

B

C

"

# Assign heights (proportions)

heights <- c(1.2, 1, 1) # Stacked bar chart occupies slightly larger proportion because it contains more samples, wider horizontally

# 2.2 Combine charts

combined_plot <- p_composition / p_box / wrap_elements(panel = corr_grob) +

plot_layout(design = layout, heights = heights)

# Step 3: Save high-resolution combined plot

ggsave("Combined_Immune_Analysis.png",

plot = combined_plot,

device = "png",

width = 10, # Optimize dimension proportions

height = 14, # Increase height to accommodate vertical layout

dpi = 600,

bg = "white")

ggsave("Combined_Immune_Analysis.pdf",

plot = combined_plot,

device = cairo_pdf,

width = 10,

height = 14,

dpi = 600)

print("Combined charts saved! Output files: Combined_Immune_Analysis.png and Combined_Immune_Analysis.pdf")

########## Step 9: Lasso Regression Builds and Validates Prediction Model #########

setwd("./diagnose1")

# Load necessary packages

library(tidyverse) # Data processing and visualization

library(caret) # Machine learning tools

library(pROC) # ROC curve analysis

library(glmnet) # Lasso regression

library(gridExtra) # Multi-plot layout

library(ggrepel) # Enhanced label positioning

library(RColorBrewer) # Color palettes

# Step 1: Data preparation and preprocessing

# Read hub gene expression data

hub_expr <- read.csv("Hub_expr.csv") %>%

column_to_rownames("Symbol") %>%

t() %>% # Transpose so samples are rows, genes are columns

as.data.frame() %>%

mutate(Group = rep(c("Control", "IRI"), each = 4)) # First 4 Control, last 4 IRI

# Step 2: Build prediction model (using Lasso regression)

set.seed(123) # Ensure reproducibility

# Prepare training data

x_train <- as.matrix(hub_expr[, 1:4]) # Hub gene expression data

y_train <- factor(hub_expr$Group, levels = c("Control", "IRI"))

# Use 10-fold cross-validation to select best lambda

cv_fit <- cv.glmnet(x_train, y_train,

family = "binomial",

alpha = 1, # Lasso regression

nfolds = 10)

# Train final model

final_model <- glmnet(x_train, y_train,

family = "binomial",

alpha = 1,

lambda = cv_fit$lambda.min)

# Extract gene importance

gene_importance <- coef(final_model) %>%

as.matrix() %>%

as.data.frame() %>%

rownames_to_column("Gene") %>%

rename(Importance = s0) %>%

filter(Gene != "(Intercept)") %>%

arrange(desc(abs(Importance)))

# Step 3: Model performance evaluation on training set

# Model prediction

train_pred <- predict(final_model, newx = x_train,

type = "response", s = cv_fit$lambda.min)

roc_train <- roc(response = as.numeric(y_train == "IRI"), predictor = train_pred)

auc_train <- round(auc(roc_train), 3) # Calculate AUC value

# Create training set prediction results data frame

train_results <- data.frame(

Actual = y_train,

Predicted_Prob = train_pred[,1],

Predicted_Class = ifelse(train_pred[,1] > 0.5, "IRI", "Control")

)

# Calculate confusion matrix

confusion_train <- confusionMatrix(factor(train_results$Predicted_Class, levels = c("Control", "IRI")),

y_train)

# Display training set performance

cat("\n=== Training Set Performance ===\n")

cat("AUC:", auc_train, "\n")

print(confusion_train)

# Step 4: Validation set data processing

# Read human validation set data

human_expr <- read.csv("GSE_5.csv") %>%

column_to_rownames("X") # Set sample names as row names

# Define mouse-human gene name mapping

gene_mapping <- c(

"Il1b" = "IL1B",

"Ripk3" = "RIPK3",

"Sting1" = "TMEM173", # STING1 human gene name

"Tnfaip3" = "TNFAIP3"

)

# Extract hub gene expression data from validation set

hub_human <- human_expr %>%

t() %>% # Transpose so samples are rows, genes are columns

as.data.frame() %>%

select(all_of(gene_mapping)) # Select mapped genes

# Restore mouse gene names

colnames(hub_human) <- names(gene_mapping)

# Add grouping information

hub_human$Group <- factor(rep(c("Control", "IRI"), times = c(7, 14)),

levels = c("Control", "IRI"))

# Check validation set data

cat("\nValidation set data dimensions:", dim(hub_human), "\n")

cat("Group distribution:\n")

print(table(hub_human$Group))

# Step 5: Model performance evaluation on validation set

x_val <- as.matrix(hub_human[, names(gene_mapping)]) # Ensure gene order matches training set

y_val <- hub_human$Group

val_pred <- predict(final_model, newx = x_val,

type = "response", s = cv_fit$lambda.min)

roc_val <- roc(response = as.numeric(y_val == "IRI"), predictor = val_pred)

auc_val <- round(auc(roc_val), 3) # Calculate AUC value

# Create validation set prediction results data frame

val_results <- data.frame(

Sample = rownames(hub_human),

Actual = y_val,

Predicted_Prob = val_pred[,1],

Predicted_Class = ifelse(val_pred[,1] > 0.5, "IRI", "Control")

)

# Calculate confusion matrix

confusion_val <- confusionMatrix(factor(val_results$Predicted_Class, levels = c("Control", "IRI")),

y_val)

# Display validation set performance

cat("\n=== Validation Set Performance ===\n")

cat("AUC:", auc_val, "\n")

print(confusion_val)

# Step 6: Result visualization

# Create custom theme

my_theme <- function(base_size = 12) {

theme_minimal(base_size = base_size) +

theme(

plot.title = element_text(hjust = 0.5, face = "bold", size = 16),

plot.subtitle = element_text(hjust = 0.5, color = "gray40", size = 12),

axis.title = element_text(size = 14),

axis.text = element_text(size = 12),

legend.position = "right",

legend.title = element_text(face = "bold"),

panel.grid.minor = element_blank(),

panel.border = element_rect(fill = NA, color = "gray80"),

strip.background = element_rect(fill = "gray90", color = NA),

strip.text = element_text(size = 12, face = "bold")

)

}

# --- Figure 1: Gene importance ---

p_importance <- gene_importance %>%

mutate(

Gene = fct_reorder(Gene, Importance),

Direction = ifelse(Importance > 0, "Positive", "Negative") # Classify based on coefficient sign

) %>%

ggplot(aes(x = Importance, y = Gene, fill = Direction)) +

geom_col(width = 0.7) +

geom_vline(xintercept = 0, linetype = "dashed", color = "gray30") +

geom_text(aes(label = sprintf("%.2f", Importance),

color = Direction),

hjust = ifelse(gene_importance$Importance > 0, -0.2, 1.2),

size = 4) +

scale_fill_manual(values = c(Negative = "tomato", Positive = "steelblue")) +

scale_color_manual(values = c(Negative = "darkred", Positive = "darkblue"), guide = "none") +

labs(title = "Hub Gene Importance in Prediction Model",

subtitle = "Positive coefficients indicate positive correlation with IRI",

x = "Regression Coefficient", y = "",

fill = "Effect Direction") +

my_theme() +

theme(legend.position = c(0.85, 0.15))

# --- Figure 2: ROC curve function ---

roc_plot <- function(roc_obj, dataset_name, auc_value) {

ggroc(roc_obj, size = 1.2, color = "navy") +

geom_segment(aes(x = 1, xend = 0, y = 0, yend = 1),

linetype = "dashed", color = "grey50") +

annotate("text", x = 0.7, y = 0.3, size = 5,

label = paste0("AUC = ", auc_value)) +

labs(title = paste0("ROC Curve (", dataset_name, ")"),

x = "Specificity", y = "Sensitivity") +

my_theme() +

coord_equal()

}

# Create ROC curve plots

p_roc_train <- roc_plot(roc_train, "Training Set", auc_train)

p_roc_val <- roc_plot(roc_val, "Validation Set", auc_val)

# --- Figure 3: Predicted probability distribution ---

# Training set predicted probability plot

p_prob_train <- train_results %>%

ggplot(aes(x = Predicted_Prob, fill = Actual)) +

geom_density(alpha = 0.7) +

geom_vline(xintercept = 0.5, linetype = "dashed", color = "red") +

scale_fill_manual(values = c(Control = "dodgerblue", IRI = "firebrick")) +

labs(title = "Predicted Probability Distribution (Training Set)",

x = "Predicted Probability of IRI", y = "Density",

fill = "Actual Class") +

my_theme()

# Validation set predicted probability plot

p_prob_val <- val_results %>%

ggplot(aes(x = Predicted_Prob, fill = Actual)) +

geom_density(alpha = 0.7) +

geom_vline(xintercept = 0.5, linetype = "dashed", color = "red") +

scale_fill_manual(values = c(Control = "dodgerblue", IRI = "firebrick")) +

labs(title = "Predicted Probability Distribution (Validation Set)",

x = "Predicted Probability of IRI", y = "Density",

fill = "Actual Class") +

my_theme()

# Step 7: Result saving and output

# Create output directories

if (!dir.exists("results")) dir.create("results")

if (!dir.exists("figures")) dir.create("figures")

# Save model

saveRDS(final_model, "results/IIRI_prediction_model.rds")

# Save gene importance

write.csv(gene_importance, "results/gene_importance.csv", row.names = FALSE)

# Save performance results

perf_results <- data.frame(

Dataset = c("Training", "Validation"),

AUC = c(auc_train, auc_val),

Sensitivity = c(confusion_train$byClass["Sensitivity"],

confusion_val$byClass["Sensitivity"]),

Specificity = c(confusion_train$byClass["Specificity"],

confusion_val$byClass["Specificity"])

)

write.csv(perf_results, "results/model_performance.csv", row.names = FALSE)

# Save prediction results

write.csv(train_results, "results/training_predictions.csv", row.names = FALSE)

write.csv(val_results, "results/validation_predictions.csv", row.names = FALSE)

# Save graphics as PNG and PDF formats

plots <- list(

importance = p_importance,

roc_train = p_roc_train,

roc_val = p_roc_val,

prob_train = p_prob_train,

prob_val = p_prob_val

)

# Save individual graphics

for (name in names(plots)) {

ggsave(paste0("figures/", name, ".png"), plot = plots[[name]],

width = 8, height = 6, dpi = 300)

ggsave(paste0("figures/", name, ".pdf"), plot = plots[[name]],

width = 8, height = 6)

}

# Create combined plots

combined_plot1 <- grid.arrange(

p_importance,

arrangeGrob(p_roc_train, p_roc_val, ncol = 2),

nrow = 2, heights = c(1.2, 1)

)

combined_plot2 <- grid.arrange(

p_prob_train,

p_prob_val,

ncol = 2

)

# Save combined graphics

ggsave("figures/combined_importance_roc.png", combined_plot1,

width = 12, height = 10, dpi = 300)

ggsave("figures/combined_importance_roc.pdf", combined_plot1,

width = 12, height = 10)

ggsave("figures/combined_probabilities.png", combined_plot2,

width = 14, height = 6, dpi = 300)

ggsave("figures/combined_probabilities.pdf", combined_plot2,

width = 14, height = 6)

# Print model performance summary

cat("\n=== Model Performance Summary ===")

cat(sprintf("\nTraining Set AUC: %.3f, Sensitivity: %.2f, Specificity: %.2f",

auc_train,

confusion_train$byClass["Sensitivity"],

confusion_train$byClass["Specificity"]))

cat(sprintf("\nValidation Set AUC: %.3f, Sensitivity: %.2f, Specificity: %.2f\n",

auc_val,

confusion_val$byClass["Sensitivity"],

confusion_val$byClass["Specificity"]))

# Graphic display

print(combined_plot1)

print(combined_plot2)

######## Optimize Model ######

setwd("./diagnose2")

# Load necessary packages

library(tidyverse)

library(caret)

library(pROC)

library(glmnet)

library(boot)

library(gridExtra)

# Set random seed

set.seed(123)

# Step 1: Data preparation and preprocessing

hub_expr <- read.csv("Hub_expr.csv") %>%

column_to_rownames("Symbol") %>%

t() %>%

as.data.frame() %>%

mutate(Group = rep(c("Control", "IRI"), each = 4)) %>%

mutate(Group = factor(Group, levels = c("Control", "IRI")))

# Data standardization

preprocess_data <- function(data) {

gene_cols <- 1:(ncol(data)-1)

data[, gene_cols] <- scale(data[, gene_cols])

return(data)

}

hub_expr_standardized <- preprocess_data(hub_expr)

cat("=== Minimal Sample Modeling Strategy ===\n")

cat("Training set: 8 samples (4 Control, 4 IRI)\n")

# Step 2: Directly use all data to train strongly regularized model

x_train <- as.matrix(hub_expr_standardized[, 1:4])

y_train <- hub_expr_standardized$Group

# Use fixed lambda value instead of cross-validation selection

cat("\n=== Training Strongly Regularized Model ===\n")

manual_lambda <- 0.1 # Use larger lambda value

final_model <- glmnet(x_train, y_train,

family = "binomial",

alpha = 1, # Lasso

lambda = manual_lambda,

standardize = FALSE)

# Step 3: Coefficient analysis

cat("\n=== Model Coefficient Analysis ===\n")

model_coef <- coef(final_model) %>%

as.matrix() %>%

as.data.frame() %>%

rownames_to_column("Gene") %>%

rename(Coefficient = s0) %>%

filter(Gene != "(Intercept)") %>%

mutate(

Importance = abs(Coefficient),

Direction = ifelse(Coefficient > 0, "Promotes IRI", "Protects from IRI"),

Selected = ifelse(Coefficient != 0, "Yes", "No")

) %>%

arrange(desc(Importance))

print(model_coef)

# Step 4: Calculate training set AUC and confidence interval

cat("\n=== Training Set Performance Evaluation ===\n")

train_pred <- predict(final_model, newx = x_train, type = "response")

roc_train <- roc(response = as.numeric(y_train == "IRI"), predictor = train_pred[,1])

auc_train <- round(auc(roc_train), 3)

# Calculate training set confidence interval

train_ci <- ci.auc(roc_train, method = "delong")

cat(sprintf("Training Set AUC: %.3f\n", auc_train))

cat(sprintf("Training Set 95%% CI (DeLong): [%.3f, %.3f]\n", train_ci[1], train_ci[3]))

# Step 5: Validation set processing

human_expr <- read.csv("GSE_5.csv") %>%

column_to_rownames("X")

gene_mapping <- c(

"Il1b" = "IL1B",

"Ripk3" = "RIPK3",

"Sting1" = "TMEM173",

"Tnfaip3" = "TNFAIP3"

)

hub_human <- human_expr %>%

t() %>%

as.data.frame() %>%

select(all_of(gene_mapping))

# Use training set mean and standard deviation for standardization

train_means <- apply(hub_expr_standardized[, 1:4], 2, mean)

train_sds <- apply(hub_expr_standardized[, 1:4], 2, sd)

hub_human_standardized <- hub_human

for(gene in names(gene_mapping)) {

hub_human_standardized[[gene]] <- (hub_human[[gene]] - train_means[gene]) / train_sds[gene]

}

hub_human_standardized$Group <- factor(rep(c("Control", "IRI"), times = c(7, 14)),

levels = c("Control", "IRI"))

# Step 6: Validation set performance evaluation

x_val <- as.matrix(hub_human_standardized[, names(gene_mapping)])

y_val <- hub_human_standardized$Group

val_pred <- predict(final_model, newx = x_val, type = "response")

roc_val <- roc(response = as.numeric(y_val == "IRI"), predictor = val_pred[,1])

auc_val <- round(auc(roc_val), 3)

# Calculate validation set confidence interval

val_ci <- ci.auc(roc_val, method = "delong")

# Step 7: Fix bootstrap function - handle single category case

cat("\n=== Validation Set Performance Bootstrap Estimation (Fixed Version) ===\n")

boot_val_function <- function(data, indices) {

boot_data <- data[indices, ]

x_boot <- as.matrix(boot_data[, 1:4])

y_boot <- boot_data$Group

# Check if both categories exist

if(length(unique(y_boot)) < 2) {

return(NA) # If only one category, return NA

}

pred_prob <- predict(final_model, newx = x_boot, type = "response")[,1]

# Safely calculate AUC

tryCatch({

roc_obj <- roc(response = as.numeric(y_boot == "IRI"), predictor = pred_prob)

return(auc(roc_obj))

}, error = function(e) {

return(NA) # If ROC calculation fails, return NA

})

}

# Execute bootstrap, increase repetitions to compensate for NA values

boot_val_results <- boot(data = hub_human_standardized,

statistic = boot_val_function,

R = 2000) # Increase repetitions

# Remove NA values

boot_auc_values <- boot_val_results$t[!is.na(boot_val_results$t)]

cat("Successfully calculated bootstrap samples:", length(boot_auc_values), "/", length(boot_val_results$t), "\n")

if(length(boot_auc_values) > 0) {

# Calculate bootstrap confidence interval

boot_val_ci <- quantile(boot_auc_values, c(0.025, 0.975), na.rm = TRUE)

cat("Bootstrap 95% CI: [", round(boot_val_ci[1], 3), ", ", round(boot_val_ci[2], 3), "]\n")

} else {

cat("Warning: All bootstrap samples failed, unable to calculate confidence interval\n")

boot_val_ci <- c(NA, NA)

}

# Step 8: Alternative method - use pROC package ci.auc for bootstrap

cat("\n=== Using pROC Package Bootstrap Confidence Interval ===\n")

roc_val_boot_ci <- ci.auc(roc_val, method = "bootstrap", boot.n = 2000, progress = "none")

cat("pROC bootstrap 95% CI: [", round(roc_val_boot_ci[1], 3), ", ", round(roc_val_boot_ci[3], 3), "]\n")

# Step 9: Univariate analysis as benchmark comparison

cat("\n=== Univariate Gene Analysis ===\n")

univariate_aucs <- data.frame(

Gene = character(),

AUC = numeric(),

stringsAsFactors = FALSE

)

for(gene in names(gene_mapping)) {

single_gene_roc <- roc(response = as.numeric(y_val == "IRI"),

predictor = hub_human_standardized[[gene]])

univariate_aucs <- rbind(univariate_aucs,

data.frame(Gene = gene, AUC = auc(single_gene_roc)))

}

univariate_aucs <- univariate_aucs %>%

arrange(desc(AUC))

print(univariate_aucs)

# Step 10: Result visualization and reporting

my_theme <- function(base_size = 12) {

theme_minimal(base_size = base_size) +

theme(

plot.title = element_text(hjust = 0.5, face = "bold"),

plot.subtitle = element_text(hjust = 0.5, color = "gray40"),

legend.position = "right"

)

}

# Coefficient plot

p_coefficients <- model_coef %>%

filter(Selected == "Yes") %>%

ggplot(aes(x = Coefficient, y = reorder(Gene, Coefficient), fill = Direction)) +

geom_col(width = 0.7) +

geom_text(aes(label = sprintf("%.3f", Coefficient)),

hjust = ifelse(model_coef$Coefficient[model_coef$Selected == "Yes"] > 0, -0.2, 1.2),

size = 4) +

scale_fill_manual(values = c("Protects from IRI" = "steelblue", "Promotes IRI" = "tomato")) +

labs(title = "Lasso Model Coefficients (Strong Regularization)",

subtitle = paste("Lambda =", manual_lambda),

x = "Standardized Coefficient", y = "") +

my_theme()

# Training set ROC curve

p_roc_train <- ggroc(roc_train, size = 1.2, color = "darkorange") +

annotate("segment", x = 1, xend = 0, y = 0, yend = 1,

linetype = "dashed", color = "grey50") +

annotate("text", x = 0.7, y = 0.3, size = 5,

label = paste0("Training AUC = ", auc_train, "\n",

"95% CI: [", round(train_ci[1], 3), ", ",

round(train_ci[3], 3), "]")) +

labs(title = "Training Set ROC Curve",

subtitle = paste("n =", nrow(hub_expr_standardized), "samples"),

x = "Specificity", y = "Sensitivity") +

my_theme() +

coord_equal()

# Validation set ROC curve

p_roc_val <- ggroc(roc_val, size = 1.2, color = "navy") +

annotate("segment", x = 1, xend = 0, y = 0, yend = 1,

linetype = "dashed", color = "grey50") +

annotate("text", x = 0.7, y = 0.3, size = 5,

label = paste0("Validation AUC = ", auc_val, "\n",

"95% CI: [", round(val_ci[1], 3), ", ",

round(val_ci[3], 3), "]")) +

labs(title = "Validation Set ROC Curve",

subtitle = paste("n =", nrow(hub_human_standardized), "samples"),

x = "Specificity", y = "Sensitivity") +

my_theme() +

coord_equal()

# Training set and validation set ROC curve comparison

p_roc_comparison <- ggroc(list(Training = roc_train, Validation = roc_val),

size = 1.2) +

scale_color_manual(values = c("Training" = "darkorange", "Validation" = "navy")) +

annotate("segment", x = 1, xend = 0, y = 0, yend = 1,

linetype = "dashed", color = "grey50") +

annotate("text", x = 0.6, y = 0.2, size = 4, color = "darkorange",

label = paste0("Training AUC = ", auc_train)) +

annotate("text", x = 0.6, y = 0.1, size = 4, color = "navy",

label = paste0("Validation AUC = ", auc_val)) +

labs(title = "Training vs Validation ROC Curves",

color = "Dataset",

x = "Specificity", y = "Sensitivity") +

my_theme() +

coord_equal()

# If bootstrap successful, add bootstrap distribution plot

if(length(boot_auc_values) > 0) {

p_boot_dist <- ggplot(data.frame(AUC = boot_auc_values), aes(x = AUC)) +

geom_histogram(binwidth = 0.05, fill = "lightblue", alpha = 0.7, color = "white") +

geom_vline(xintercept = auc_val, linetype = "dashed", color = "red", size = 1) +

geom_vline(xintercept = boot_val_ci[1], linetype = "dotted", color = "blue") +

geom_vline(xintercept = boot_val_ci[2], linetype = "dotted", color = "blue") +

annotate("text", x = auc_val, y = Inf,

label = paste0("AUC = ", auc_val),

vjust = 2, hjust = -0.1, color = "red", fontface = "bold") +

labs(title = "Bootstrap AUC Distribution",

subtitle = paste("Based on", length(boot_auc_values), "successful samples"),

x = "AUC", y = "Frequency") +

my_theme()

}

# Univariate comparison plot

p_univariate <- univariate_aucs %>%

ggplot(aes(x = AUC, y = reorder(Gene, AUC))) +

geom_col(fill = "darkgreen", alpha = 0.7, width = 0.7) +

geom_text(aes(label = sprintf("%.3f", AUC)), hjust = -0.2, size = 4) +

geom_vline(xintercept = auc_val, linetype = "dashed", color = "red", size = 1) +

geom_vline(xintercept = auc_train, linetype = "dashed", color = "darkorange", size = 1) +

annotate("text", x = auc_val, y = 2.5,

label = paste("Model Validation AUC =", auc_val),

hjust = -0.1, color = "red", fontface = "bold") +

annotate("text", x = auc_train, y = 1.5,

label = paste("Model Training AUC =", auc_train),

hjust = -0.1, color = "darkorange", fontface = "bold") +

labs(title = "Single Gene vs. Multivariate Model Performance",

subtitle = "Comparison of univariate AUCs with full model performance",

x = "AUC", y = "") +

my_theme() +

xlim(0, 1)

# Step 11: Detailed report

cat("\n=== Minimal Sample Modeling Results Report ===\n")

cat("Data characteristics:\n")

cat("- Training set: 8 samples (4 Control, 4 IRI)\n")

cat("- Validation set: 21 samples (7 Control, 14 IRI)\n")

cat("- Number of features: 4 genes\n\n")

cat("Modeling strategy:\n")

cat("1. Use strong regularization (lambda =", manual_lambda, ") to prevent overfitting\n")

cat("2. Avoid internal cross-validation (insufficient sample size)\n")

cat("3. Focus on validation set performance\n")

cat("4. Provide univariate analysis as benchmark\n\n")

cat("Model coefficients:\n")

for(i in 1:nrow(model_coef)) {

if(model_coef$Selected[i] == "Yes") {

cat(sprintf("- %s: %.4f (%s)\n",

model_coef$Gene[i],

model_coef$Coefficient[i],

model_coef$Direction[i]))

} else {

cat(sprintf("- %s: Compressed to 0 by Lasso (feature selection)\n", model_coef$Gene[i]))

}

}

cat("\nPerformance results:\n")

cat(sprintf("Training Set AUC: %.3f (95%% CI: [%.3f, %.3f])\n", auc_train, train_ci[1], train_ci[3]))

cat(sprintf("Validation Set AUC: %.3f (95%% CI: [%.3f, %.3f])\n", auc_val, val_ci[1], val_ci[3]))

cat(sprintf("Validation Set 95%% CI (pROC Bootstrap): [%.3f, %.3f]\n",

roc_val_boot_ci[1], roc_val_boot_ci[3]))

if(length(boot_auc_values) > 0) {

cat(sprintf("Validation Set 95%% CI (Manual Bootstrap): [%.3f, %.3f]\n",

boot_val_ci[1], boot_val_ci[2]))

}

cat("\nSingle gene performance comparison:\n")

for(i in 1:nrow(univariate_aucs)) {

cat(sprintf("- %s: AUC = %.3f\n", univariate_aucs$Gene[i], univariate_aucs$AUC[i]))

}

# Step 12: Prediction result analysis

val_results <- data.frame(

Sample = rownames(hub_human_standardized),

Actual = y_val,

Predicted_Prob = val_pred[,1],

Predicted_Class = ifelse(val_pred[,1] > 0.5, "IRI", "Control")

)

# Calculate confusion matrix

confusion_val <- confusionMatrix(factor(val_results$Predicted_Class, levels = c("Control", "IRI")),

y_val)

cat("\nValidation set classification performance:\n")

print(confusion_val$table)

cat(sprintf("Accuracy: %.3f\n", confusion_val$overall["Accuracy"]))

cat(sprintf("Sensitivity: %.3f\n", confusion_val$byClass["Sensitivity"]))

cat(sprintf("Specificity: %.3f\n", confusion_val$byClass["Specificity"]))

# Step 13: Save results

if (!dir.exists("results")) dir.create("results")

if (!dir.exists("figures")) dir.create("figures")

# Save model and coefficients

saveRDS(final_model, "results/robust_IIRI_model.rds")

write.csv(model_coef, "results/model_coefficients.csv", row.names = FALSE)

write.csv(val_results, "results/validation_predictions.csv", row.names = FALSE)

# Save performance summary

performance_summary <- data.frame(

Dataset = c("Training", "Validation"),

AUC = c(auc_train, auc_val),

AUC_CI_Lower_Delong = c(train_ci[1], val_ci[1]),

AUC_CI_Upper_Delong = c(train_ci[3], val_ci[3]),

AUC_CI_Lower_Bootstrap = c(NA, roc_val_boot_ci[1]),

AUC_CI_Upper_Bootstrap = c(NA, roc_val_boot_ci[3]),

Accuracy = c(NA, confusion_val$overall["Accuracy"]),

Sensitivity = c(NA, confusion_val$byClass["Sensitivity"]),

Specificity = c(NA, confusion_val$byClass["Specificity"])

)

write.csv(performance_summary, "results/performance_summary.csv", row.names = FALSE)

# Step 14: Save graphics as PDF format, 600dpi

cat("\n=== Save Graphics as PDF Format (600 dpi) ===\n")

# Save all graphics as PDF

ggsave("figures/model_coefficients.pdf", p_coefficients, width = 8, height = 6, dpi = 600)

ggsave("figures/training_roc.pdf", p_roc_train, width = 8, height = 6, dpi = 600)

ggsave("figures/validation_roc.pdf", p_roc_val, width = 8, height = 6, dpi = 600)

ggsave("figures/roc_comparison.pdf", p_roc_comparison, width = 8, height = 6, dpi = 600)

ggsave("figures/univariate_comparison.pdf", p_univariate, width = 8, height = 6, dpi = 600)

if(length(boot_auc_values) > 0) {

ggsave("figures/bootstrap_distribution.pdf", p_boot_dist, width = 8, height = 6, dpi = 600)

}

# Create comprehensive graphic panel

if(length(boot_auc_values) > 0) {

# Create large panel containing all graphics

p_all <- grid.arrange(p_coefficients, p_roc_comparison, p_boot_dist, p_univariate,

ncol = 2,

layout_matrix = rbind(c(1, 2), c(3, 4)),

top = "Comprehensive Analysis of Small Sample Modeling")

} else {

p_all <- grid.arrange(p_coefficients, p_roc_comparison, p_univariate,

ncol = 2,

layout_matrix = rbind(c(1, 2), c(1, 3)),

top = "Comprehensive Analysis of Small Sample Modeling")

}

# Save comprehensive panel

ggsave("figures/comprehensive_analysis.pdf", p_all, width = 16, height = 12, dpi = 600)

cat("Graphics saved as PDF format (600 dpi):\n")

cat("- model_coefficients.pdf: Model coefficient plot\n")

cat("- training_roc.pdf: Training set ROC curve\n")

cat("- validation_roc.pdf: Validation set ROC curve\n")

cat("- roc_comparison.pdf: Training set vs validation set ROC curve comparison\n")

cat("- univariate_comparison.pdf: Univariate comparison plot\n")

if(length(boot_auc_values) > 0) {

cat("- bootstrap_distribution.pdf: Bootstrap distribution plot\n")

}

cat("- comprehensive_analysis.pdf: Comprehensive analysis panel\n")

# Step 15: Create final report

cat("\n=== Final Recommendations ===\n")

cat("✓ Use strongly regularized model to avoid overfitting\n")

cat(sprintf("✓ Training Set AUC = %.3f, Validation Set AUC = %.3f\n", auc_train, auc_val))

cat("✓ Validation set performance provides true estimate of model generalization ability\n")

cat("✓ Multiple confidence interval methods enhance result credibility\n")

cat("✓ Considering minimal sample size, results should be regarded as preliminary findings\n")

cat("✓ Recommend validating these results on larger samples\n")

# Display graphics

if(length(boot_auc_values) > 0) {

grid.arrange(p_coefficients, p_roc_comparison, p_boot_dist, p_univariate, ncol = 2,

layout_matrix = rbind(c(1, 2), c(3, 4)))

} else {

grid.arrange(p_coefficients, p_roc_comparison, p_univariate, ncol = 2,

layout_matrix = rbind(c(1, 2), c(1, 3)))

}

############## Step 10: Therapeutic Target Prediction ###########

setwd("./drug")

# Load necessary packages

library(tidyverse) # Data manipulation and visualization

library(visNetwork) # Interactive network plots

library(readr) # Efficient data reading

library(ggrepel) # Avoid label overlap

library(scales) # Color scales

library(RColorBrewer)# Color palettes

# Step 1: Read data

# Read hub gene file

hub_genes <- read_csv("Hub Gene_human.csv", col_types = cols()) %>%

pull(Gene) %>%

unique()

head(hub_genes)

# Read DGIdb result file

dgidb_data <- read_tsv("DGIdb_results.tsv", col_types = cols()) %>%

# Standardize column names

rename(

gene = `gene`,

drug = `drug`,

regulatory_approval = `regulatory approval`,

indication = `indication`,

interaction_score = `interaction score`

) %>%

# Create simplified approval status column

mutate(approval_status = if_else(regulatory_approval == "Approved", "Approved", "Experimental")) %>%

# Filter to only keep hub genes

filter(gene %in% hub_genes) %>%

# Filter out missing values

filter(!is.na(gene), !is.na(drug))

# Check data

cat("Hub genes count:", length(hub_genes), "\n")

cat("Hub genes found in DGIdb:", toString(unique(dgidb_data$gene)), "\n")

cat("Drugs count:", n_distinct(dgidb_data$drug), "\n")

cat("Total interactions:", nrow(dgidb_data), "\n")

# Step 2: Data preprocessing and statistics

# Calculate drug statistical information for each gene

gene_stats <- dgidb_data %>%

group_by(gene) %>%

summarize(

total_drugs = n(),

approved_drugs = sum(approval_status == "Approved"),

experimental_drugs = sum(approval_status == "Experimental"),

mean_score = mean(interaction_score, na.rm = TRUE),

max_score = max(interaction_score, na.rm = TRUE)

) %>%

ungroup() %>%

# Sort by total number of drugs

arrange(desc(total_drugs)) %>%

# Create sorting factor

mutate(gene = factor(gene, levels = unique(gene)))

# Calculate score ranking for each gene-drug

dgidb_data <- dgidb_data %>%

group_by(gene) %>%

mutate(score_rank = dense_rank(desc(interaction_score))) %>%

ungroup()

# Step 3: Draw advanced bar plot (English labels)

# Create long format data for stacked bar plot

stack_data <- gene_stats %>%

select(gene, approved_drugs, experimental_drugs) %>%

pivot_longer(

cols = -gene,

names_to = "drug_type",

values_to = "count"

) %>%

mutate(drug_type = recode(drug_type,

"approved_drugs" = "Approved Drugs",

"experimental_drugs" = "Experimental Drugs"))

# Create stacked bar plot

bar_plot <- ggplot(stack_data, aes(x = gene, y = count, fill = drug_type)) +

geom_bar(stat = "identity", position = "stack", width = 0.7) +

geom_text(aes(label = count),

position = position_stack(vjust = 0.5),

color = "white", size = 3.5, fontface = "bold") +

geom_text(

data = gene_stats,

aes(x = gene, y = total_drugs + 0.5, label = paste0("Avg score: ", round(mean_score, 2))),

inherit.aes = FALSE, # Avoid aes inheritance error

size = 3.2, color = "#333333", vjust = -0.5

) +

# Beautify graphic

scale_fill_manual(values = c("Approved Drugs" = "#2ca25f", "Experimental Drugs" = "#e34a33")) +

scale_y_continuous(expand = expansion(mult = c(0, 0.1))) +

labs(

title = "Drug Interactions for Hub Genes",

subtitle = "Based on DGIdb Predictions",

x = "Hub Genes",

y = "Number of Drugs",

fill = "Drug Type",

caption = "Data source: DGIdb | Top labels show average interaction score"

) +

theme_minimal(base_size = 12) +

theme(

plot.title = element_text(size = 18, face = "bold", hjust = 0.5, margin = margin(b = 10)),

plot.subtitle = element_text(size = 14, hjust = 0.5, color = "#555555", margin = margin(b = 20)),

axis.title = element_text(size = 12, face = "bold"),

axis.text.x = element_text(angle = 45, hjust = 1, size = 11, face = "bold"),

axis.text.y = element_text(size = 10),

legend.position = "top",

legend.title = element_text(face = "bold"),

legend.text = element_text(size = 10),

panel.grid.major.x = element_blank(),

panel.grid.minor.y = element_blank(),

plot.caption = element_text(color = "#777777", size = 9, margin = margin(t = 15)),

plot.margin = margin(20, 20, 20, 20)

)

# Display bar plot

print(bar_plot)

# Step 4: Prepare network plot data (English labels)

# Create node data frame

genes <- unique(dgidb_data$gene)

drugs <- unique(dgidb_data$drug)

# Gene nodes

gene_nodes <- gene_stats %>%

mutate(

id = gene,

label = gene,

group = "Hub Gene",

value = total_drugs * 1.5,

title = paste0(

"<p><b>", gene, "</b>",

"<br>Total drugs: ", total_drugs,

"<br>Approved: ", approved_drugs,

"<br>Experimental: ", experimental_drugs,

"<br>Avg score: ", round(mean_score, 2)

),

shape = "dot",

color = "#1f78b4",

shadow = TRUE,

# Fix solution: Add missing color attribute to make list length 3

font = list(

size = 24,

face = "bold",

color = "black" # Add font color attribute

)

) %>%

select(id, label, group, value, title, shape, color)

# Drug nodes

drug_nodes <- dgidb_data %>%

mutate(

id = drug,

label = str_trunc(drug, 20), # Truncate long drug names

group = "Drug",

value = sqrt(interaction_score) * 2, # Node size based on score

title = paste0(

"<p><b>", drug, "</b>",

"<br>Gene: ", gene,

"<br>Status: ", regulatory_approval,

"<br>Indication: ", ifelse(is.na(indication), "N/A", str_trunc(indication, 50)),

"<br>Score: ", round(interaction_score, 2)),

shape = "square",

color = ifelse(approval_status == "Approved", "#33a02c", "#fb9a99"), # Green=approved, pink=experimental

shadow = TRUE

) %>%

select(id, label, group, value, title, shape, color) %>%

distinct(id, .keep_all = TRUE) # Ensure each drug has only one node

# Merge nodes

all_nodes <- bind_rows(gene_nodes, drug_nodes)

# Create edge data frame

edges <- dgidb_data %>%

mutate(

from = gene,

to = drug,

title = paste0(

"Score: ", round(interaction_score, 2),

"<br>Status: ", regulatory_approval

),

value = interaction_score * 0.1, # Edge width

color = ifelse(approval_status == "Approved", "#2ca25f", "#de2d26"), # Green=approved, red=experimental

dashes = ifelse(approval_status == "Approved", FALSE, TRUE) # Experimental drugs use dashed lines

) %>%

select(from, to, title, value, color, dashes)

# Step 5: Draw interactive network plot (English labels)

# Create interactive network plot

network_plot <- visNetwork(all_nodes, edges, width = "100%", height = "800px") %>%

visOptions(

highlightNearest = list(enabled = TRUE, degree = 1, hover = TRUE),

nodesIdSelection = list(enabled = TRUE, style = "width: 200px; height: 30px;"),

selectedBy = list(variable = "group", style = "width: 150px; height: 30px;")

) %>%

visGroups(groupname = "Hub Gene", shape = "dot", color = list(background = "#1f78b4", border = "#0d3c61")) %>%

visGroups(groupname = "Drug", shape = "square") %>%

visLayout(randomSeed = 123) %>% # Fixed layout

visPhysics(

solver = "forceAtlas2Based",

forceAtlas2Based = list(gravitationalConstant = -100, avoidOverlap = 0.5)

) %>%

visInteraction(

navigationButtons = TRUE,

tooltipDelay = 100,

keyboard = TRUE,

dragView = TRUE,

zoomView = TRUE

) %>%

visLegend(

position = "right",

stepY = 50,

useGroups = TRUE,

main = "Legend"

) %>%

visEdges(

smooth = list(enabled = TRUE, type = "dynamic", roundness = 0.5),

arrows = list(to = list(enabled = TRUE, scaleFactor = 0.5)),

scaling = list(min = 1, max = 10)

) %>%

visNodes(

borderWidth = 2,

borderWidthSelected = 4

) %>%

addFontAwesome() %>%

visConfigure(enabled = TRUE) # Add configuration options

# Display network plot

network_plot

# Step 6: Save high-quality results

# Save bar plot (high-quality PNG and PDF)

ggsave("HubGenes_DrugInteractions.png", plot = bar_plot,

width = 10, height = 7, dpi = 600, bg = "white")

ggsave("HubGenes_DrugInteractions.pdf", plot = bar_plot,

width = 10, height = 7, device = cairo_pdf)

# Save interactive network plot

visSave(network_plot, file = "HubGenes_DrugNetwork.html", selfcontained = TRUE)

# Save data as CSV

write_csv(gene_stats, "HubGenes_SummaryStats.csv")

write_csv(dgidb_data, "HubGenes_DGIdbDetails.csv")

# Output session information

cat("\n===== Analysis Completed =====\n")

sessionInfo()

###### Modified Code Static Network Plot #####

# Load necessary packages

library(tidyverse) # Data manipulation and visualization

library(visNetwork) # Interactive network plots

library(readr) # Efficient data reading

library(ggrepel) # Avoid label overlap

library(scales) # Color scales

library(RColorBrewer)# Color palettes

library(igraph) # Network plot analysis and visualization

library(ggraph) # ggplot2-based network plots

library(patchwork) # Image composition

# Select top 3 drugs per gene (sorted by score)

simplified_data <- dgidb_data %>%

group_by(gene) %>%

arrange(desc(interaction_score)) %>%

slice_head(n = 3) %>% # Keep top 3 per gene

ungroup()

# Create igraph object

graph_data <- graph_from_data_frame(

simplified_data %>% select(gene, drug, approval_status, interaction_score),

directed = FALSE

)

# Set node attributes

V(graph_data)$type <- ifelse(V(graph_data)$name %in% hub_genes, "Gene", "Drug")

V(graph_data)$color <- case_when(

V(graph_data)$type == "Gene" ~ "#1f78b4", # Blue represents genes

V(graph_data)$type == "Drug" &

(simplified_data$approval_status[match(V(graph_data)$name, simplified_data$drug)] == "Approved") ~ "#33a02c", # Green represents approved drugs

TRUE ~ "#fb9a99" # Pink represents experimental drugs

)

# Set node size

V(graph_data)$size <- ifelse(

V(graph_data)$type == "Gene",

15, # Gene nodes larger

sqrt(simplified_data$interaction_score[match(V(graph_data)$name, simplified_data$drug)] * 5) # Drug node size based on score

)

# Set edge attributes

E(graph_data)$width <- simplified_data$interaction_score * 0.5

E(graph_data)$linetype <- ifelse(

simplified_data$approval_status == "Approved",

"solid", # Solid line represents approved

"dashed" # Dashed line represents experimental

)

# Create static network plot

static_network <- ggraph(graph_data, layout = "fr") + # Fruchterman-Reingold layout

geom_edge_link(

aes(linetype = linetype, width = width),

color = "grey70",

alpha = 0.8,

show.legend = TRUE

) +

geom_node_point(

aes(color = color, size = size),

alpha = 0.95

) +

geom_node_text(

aes(label = ifelse(type == "Gene", name, "")), # Only mark genes

size = 5,

nudge_y = 0.1,

fontface = "bold",

check_overlap = TRUE

) +

geom_node_label(

aes(label = ifelse(type == "Drug", name, ""), # Drugs use label boxes

fill = ifelse(type == "Drug", alpha("white", 0.7), NA)),

size = 3,

repel = TRUE,

segment.color = "grey50",

box.padding = 0.25

) +

scale_edge_width(range = c(0.5, 3), name = "Interaction Score") +

scale_color_identity() +

scale_size_identity() +

scale_edge_linetype_manual(

values = c("dashed" = "dashed", "solid" = "solid"),

labels = c("Experimental", "Approved"),

name = "Approval Status"

) +

guides(

color = "none",

size = "none",

linetype = guide_legend(

override.aes = list(edge_width = 2, edge_color = "black")

)

) +

labs(

title = "Hub Genes and Drug Interactions",

subtitle = "Top 3 drugs per gene by interaction score",

caption = "Blue nodes: Hub genes | Green nodes: Approved drugs | Pink nodes: Experimental drugs"

) +

theme_void(base_size = 14) +

theme(

plot.title = element_text(size = 20, face = "bold", hjust = 0.5),

plot.subtitle = element_text(size = 15, hjust = 0.5, margin = margin(b = 15)),

plot.caption = element_text(size = 10, color = "grey40", margin = margin(t = 15)),

legend.position = "bottom",

legend.title = element_text(face = "bold"),

legend.text = element_text(size = 10),

plot.margin = margin(20, 20, 20, 20),

panel.background = element_rect(fill = "white", color = NA)

)

# Display static network plot

print(static_network)

# Step 5: Save all results

# Save bar plot (high-quality PNG and PDF)

ggsave("HubGenes_DrugInteractions.png", plot = bar_plot,

width = 10, height = 7, dpi = 600, bg = "white")

ggsave("HubGenes_DrugInteractions.pdf", plot = bar_plot,

width = 10, height = 7, device = cairo_pdf)

# Save static network plot (PDF and PNG)

ggsave("HubGenes_SimplifiedNetwork.png", plot = static_network,

width = 15, height = 9, dpi = 600, bg = "white")

ggsave("HubGenes_SimplifiedNetwork.pdf", plot = static_network,

width = 15, height = 9, device = cairo_pdf)

# Save data as CSV

write_csv(gene_stats, "HubGenes_SummaryStats.csv")

write_csv(dgidb_data, "HubGenes_DGIdbDetails.csv")

# Output session information

cat("\n===== Analysis Completed =====\n")

sessionInfo()

############# Gene-Drug Network Plot #########

setwd("./drug")

# Load necessary packages

library(dplyr)

library(tidyr)

library(igraph)

library(ggraph)

library(ggplot2)

library(scales)

library(viridis)

# Step 1: Read data

dgidb_data <- read.delim("DGIdb_results.tsv", sep = "\t", header = TRUE, stringsAsFactors = FALSE)

# Step 2: Data preprocessing

cleaned_data <- dgidb_data %>%

filter(!is.na(drug) & drug != "") %>%

mutate(approval_status = ifelse(regulatory.approval == "Approved", "Approved", "Not Approved")) %>%

mutate(drug_id = paste0("D_", row_number())) %>%

select(gene, drug, drug_id, interaction.score, approval_status)

# Step 3: Create node data - add color classification

# Gene nodes

gene_nodes <- cleaned_data %>%

distinct(gene) %>%

mutate(

node_id = gene,

node_type = "Gene",

node_group = "Gene", # Add grouping information

size = 5,

color = "#1f77b4" # Gene nodes uniformly blue

) %>%

select(node_id, node_type, size, node_group, color)

# Drug nodes - assign colors based on approval status

drug_nodes <- cleaned_data %>%

distinct(drug_id, drug, approval_status) %>%

mutate(

node_id = drug_id,

node_type = "Drug",

size = ifelse(approval_status == "Approved", 8, 6),

node_group = ifelse(approval_status == "Approved", "Approved Drug", "Not Approved Drug"),

color = ifelse(approval_status == "Approved", "#2ca02c", "#fb9a99") # Approved drugs green, unapproved pink

) %>%

select(node_id, node_type, size, label = drug, node_group, color)

# Merge all nodes

all_nodes <- bind_rows(gene_nodes, drug_nodes) %>%

mutate(node_id = as.character(node_id))

# Step 4: Create edge data

edges <- cleaned_data %>%

mutate(

from = gene,

to = drug_id,

width = rescale(interaction.score, to = c(1, 5)),

score_label = sprintf("%.2f", interaction.score)

) %>%

select(from, to, width, score_label)

# Step 5: Create network plot object

net <- graph_from_data_frame(

d = edges,

vertices = all_nodes,

directed = FALSE

)

# Step 6: Network visualization

# Corrected network visualization code

set.seed(123) # Ensure reproducible layout

# Create unified label column

all_nodes <- all_nodes %>%

mutate(

plot_label = case_when(

node_type == "Gene" ~ node_id,

node_type == "Drug" ~ label

)

)

# Update network plot object

net <- graph_from_data_frame(

d = edges,

vertices = all_nodes,

directed = FALSE

)

ggraph(net, layout = "fr") + # Fruchterman-Reingold layout algorithm

# Draw edges (transparency 0.7, graded by width)

geom_edge_link(

aes(edge_width = width),

alpha = 0.7,

color = "grey50",

show.legend = TRUE

) +

# Draw nodes (distinguished by type and approval status)

geom_node_point(

aes(

size = size,

shape = node_type,

color = color # Directly use predefined colors

),

show.legend = TRUE

) +

# Add all node labels

geom_node_text(

aes(label = plot_label), # Use unified label column

size = 3,

repel = TRUE,

max.overlaps = 20,

bg.color = "white", # Add white background to improve readability

bg.r = 0.15

) +

# Custom scales

scale_size_continuous(

name = "Node Size",

range = c(3, 8),

breaks = c(5, 6, 8),

labels = c("Gene", "Not Approved Drug", "Approved Drug")

) +

scale_shape_manual(

name = "Node Type",

values = c("Gene" = 16, "Drug" = 15),

labels = c("Gene", "Drug")

) +

scale_color_identity( # Use predefined colors

name = "Node Type",

guide = "legend",

labels = c("Gene", "Approved Drug", "Not Approved Drug"),

breaks = c("#1f77b4", "#2ca02c", "#fb9a99")

) +

scale_edge_width_continuous(

name = "Interaction Score",

range = c(0.5, 3)

) +

# Theme settings

theme_void() +

theme(

legend.position = "right",

plot.title = element_text(hjust = 0.5, size = 16, face = "bold"),

plot.subtitle = element_text(hjust = 0.5, size = 12),

legend.box = "vertical",

legend.text = element_text(size = 10),

legend.title = element_text(size = 11, face = "bold")

) +

# Add title

labs(

title = "Gene-Drug Interaction Network",

subtitle = "Data from DGIdb Database Analysis",

caption = "Edge width proportional to interaction score\nNode size and color indicate regulatory approval status"

)

# Step 7: Save results

ggsave("gene_drug_network.png", width = 12, height = 10, dpi = 300)

ggsave("gene_drug_network.pdf", width = 12, height = 10, dpi = 300)

# Step 8: Combine images

# Load necessary packages

library(patchwork)

library(cowplot)

library(ggplot2)

library(dplyr)

library(tidyr)

library(igraph)

library(ggraph)

library(scales)

# Create gene-drug network plot

# Read data

dgidb_data <- read.delim("DGIdb_results.tsv", sep = "\t", header = TRUE, stringsAsFactors = FALSE)

# Data preprocessing

cleaned_data <- dgidb_data %>%

filter(!is.na(drug) & drug != "") %>%

mutate(approval_status = ifelse(regulatory.approval == "Approved", "Approved", "Not Approved")) %>%

mutate(drug_id = paste0("D_", row_number())) %>%

select(gene, drug, drug_id, interaction.score, approval_status)

# Create node data

gene_nodes <- cleaned_data %>%

distinct(gene) %>%

mutate(

node_id = gene,

node_type = "Gene",

node_group = "Gene",

size = 5,

color = "#1f77b4",

label = gene

) %>%

select(node_id, node_type, size, node_group, color, label)

drug_nodes <- cleaned_data %>%

distinct(drug_id, drug, approval_status) %>%

mutate(

node_id = drug_id,

node_type = "Drug",

size = ifelse(approval_status == "Approved", 8, 6),

node_group = ifelse(approval_status == "Approved", "Approved Drug", "Not Approved Drug"),

color = ifelse(approval_status == "Approved", "#2ca02c", "#fb9a99"),

label = drug

) %>%

select(node_id, node_type, size, label, node_group, color)

all_nodes <- bind_rows(gene_nodes, drug_nodes) %>%

mutate(node_id = as.character(node_id))

# Create edge data

edges <- cleaned_data %>%

mutate(

from = gene,

to = drug_id,

width = rescale(interaction.score, to = c(1, 5)),

score_label = sprintf("%.2f", interaction.score)

) %>%

select(from, to, width, score_label)

# Create network plot object

net <- graph_from_data_frame(

d = edges,

vertices = all_nodes,

directed = FALSE

)

# Create gene-drug network plot (simplified labels)

gene_drug_network_plot <- ggraph(net, layout = "fr") +

geom_edge_link(

aes(edge_width = width),

alpha = 0.7,

color = "grey50",

show.legend = FALSE

) +

geom_node_point(

aes(size = size, color = color),

show.legend = FALSE

) +

# Only show gene labels and important drug labels

geom_node_text(

aes(label = ifelse(node_type == "Gene" | size > 7, label, "")),

size = 3.5,

repel = TRUE,

max.overlaps = 50,

bg.color = "white",

bg.r = 0.15

) +

scale_size_identity() +

scale_color_identity() +

scale_edge_width_continuous(range = c(0.5, 3)) +

labs(

title = "Comprehensive Drug-Gene Interaction Network"

) +

theme_void() +

theme(

plot.title = element_text(hjust = 0.5, size = 16, face = "bold"),

plot.margin = margin(10, 10, 10, 10)

)

# Combine charts - vertical side-by-side layout

# Optimize display of each chart

bar_plot_optimized <- bar_plot +

theme(

plot.title = element_text(size = 18, face = "bold", hjust = 0.5),

axis.title = element_text(size = 14),

axis.text.x = element_text(size = 12, angle = 45, hjust = 1),

axis.text.y = element_text(size = 12),

legend.title = element_text(size = 14),

legend.text = element_text(size = 12),

legend.position = "bottom"

) +

labs(title = "Drug Interactions by Hub Gene")

static_network_optimized <- static_network +

theme(

plot.title = element_text(size = 18, face = "bold", hjust = 0.5),

plot.margin = margin(10, 20, 10, 20)

) +

labs(title = "Top Drug Interactions")

gene_drug_optimized <- gene_drug_network_plot +

theme(

plot.title = element_text(size = 18, face = "bold", hjust = 0.5),

plot.margin = margin(10, 20, 10, 20)

) +

labs(title = "Comprehensive Drug Network")

# Create combined charts - vertical side-by-side layout

combined_plot <- ggdraw() +

# Main title

draw_text("Comprehensive Drug Target Analysis",

x = 0.5, y = 0.98, size = 22, fontface = "bold") +

# 1. Top: Bar chart (height 25%)

draw_plot(bar_plot_optimized,

x = 0.05, y = 0.70,

width = 0.90, height = 0.25) +

# 2. Middle: Simplified network plot (height 35%)

draw_plot(static_network_optimized,

x = 0.05, y = 0.35,

width = 0.90, height = 0.35) +

# 3. Bottom: Complete network plot (height 35%)

draw_plot(gene_drug_optimized,

x = 0.05, y = 0.00,

width = 0.90, height = 0.35) +

# Subplot labels

draw_text("A", x = 0.03, y = 0.95, size = 20, fontface = "bold", color = "black") +

draw_text("B", x = 0.03, y = 0.68, size = 20, fontface = "bold", color = "black") +

draw_text("C", x = 0.03, y = 0.33, size = 20, fontface = "bold", color = "black") +

# Legend description

draw_label("Color Legend:\n• Genes: Blue\n• Approved Drugs: Green\n• Experimental Drugs: Pink",

x = 0.82, y = 0.12, size = 14, hjust = 0, color = "black")

# Save combined charts

ggsave("Combined_Drug_Target_Analysis.png", combined_plot,

width = 16, height = 20, dpi = 600, bg = "white")

ggsave("Combined_Drug_Target_Analysis.pdf", combined_plot,

width = 16, height = 20, device = cairo_pdf)

cat("Combined plot successfully created with vertical layout.\n")

####### Step 11: Convert Mouse Hub Genes to Human Homologs ########

setwd("./Human Hub Gene")

# Install and load necessary R packages

if (!require("biomaRt")) install.packages("biomaRt")

library(biomaRt)

packageVersion("biomaRt")

# Step 1: Read mouse hub gene list

mouse_genes <- read.csv("Hub Gene.csv", header = TRUE)

gene_list <- mouse_genes$Gene

print("Original mouse gene list:")

print(gene_list)

# Step 2: Define reliable mirror site list

mirror_sites <- c(

"https://www.ensembl.org", # Main site

"https://asia.ensembl.org", # Asia mirror

"https://useast.ensembl.org", # US East mirror

"https://ensembl.org" # General domain

)

# Step 3: Try different mirror sites

success <- FALSE

for (host in mirror_sites) {

tryCatch({

cat("Trying mirror site:", host, "\n")

# Create Mart object

ensembl_mouse <- useMart(

"ensembl",

dataset = "mmusculus_gene_ensembl",

host = host

)

ensembl_human <- useMart(

"ensembl",

dataset = "hsapiens_gene_ensembl",

host = host

)

# Perform gene homolog conversion

homologs <- getLDS(

attributes = c("mgi_symbol", "ensembl_gene_id"),

filters = "mgi_symbol",

values = gene_list,

mart = ensembl_mouse,

attributesL = c("hgnc_symbol", "ensembl_gene_id"),

martL = ensembl_human,

uniqueRows = TRUE

)

# Rename result columns

colnames(homologs) <- c("Mouse_Gene", "Mouse_Ensembl_ID",

"Human_Gene", "Human_Ensembl_ID")

# Check and process results

matched_genes <- unique(homologs$Mouse_Gene)

unmatched <- setdiff(gene_list, matched_genes)

# Output results

cat("\nConversion successful! Using mirror site:", host, "\n")

print("Conversion results:")

print(homologs)

if (length(unmatched) > 0) {

print("The following genes did not find human homologs:")

print(unmatched)

}

# Save results as CSV file

write.csv(homologs, "Human_Homologs_Results.csv", row.names = FALSE)

cat("Results saved to Human_Homologs_Results.csv\n")

success <- TRUE

break # Break loop if successful

}, error = function(e) {

cat("Site", host, "connection failed:", conditionMessage(e), "\n\n")

})

}

# Step 4: If all mirrors fail, provide alternative solutions

if (!success) {

cat("\nAll mirror sites failed to connect. Please try the following solutions:\n")

cat("1. Check network connection, ensure access to Ensembl database\n")

cat("2. Try again after waiting for a while (server may be temporarily maintained)\n")

cat("3. Use the following manual mapping results (based on latest ENSEMBL database):\n")

# Provide known mapping relationships

manual_mapping <- data.frame(

Mouse_Gene = c("Il1b", "Ripk3", "Sting1", "Tnfaip3"),

Mouse_Ensembl_ID = c("ENSMUSG00000027398", "ENSMUSG00000025482",

"ENSMUSG00000027978", "ENSMUSG00000030669"),

Human_Gene = c("IL1B", "RIPK3", "STING1", "TNFAIP3"),

Human_Ensembl_ID = c("ENSG00000125538", "ENSG00000129465",

"ENSG00000184584", "ENSG00000118503")

)

print(manual_mapping)

write.csv(manual_mapping, "Human_Homologs_Manual.csv", row.names = FALSE)

cat("Manual mapping results saved to Human_Homologs_Manual.csv\n")

}
